# Differentially methylated loci in NAFLD cirrhosis are associated with key signaling pathways

**DOI:** 10.1186/s13148-018-0525-9

**Published:** 2018-07-13

**Authors:** Glenn S. Gerhard, Ivana Malenica, Lorida Llaci, Xin Chu, Anthony T. Petrick, Christopher D. Still, Johanna K. DiStefano

**Affiliations:** 1 Temple University School of Medicine, Philadelphia, PA 19140 USA; 2 Translational Genomics Research Institute, 445 N 5th St, Phoenix, AZ 85004 USA; 3 Geisinger Obesity Institute, Danville, PA 17822 USA

**Keywords:** Nonalcoholic fatty liver disease, Nonalcoholic steatohepatitis, Liver fibrosis, DNA methylation, Epigenetics, Severe obesity, Bariatric surgery

## Abstract

Altered DNA methylation events contribute to the pathogenesis and progression of metabolic disorders, including nonalcoholic fatty liver disease (NAFLD). Investigations of global DNA methylation patterns in liver biopsies representing severe NAFLD fibrosis have been limited. We used the HumanMethylation 450K BeadChip to analyze genome-wide methylation in patients with biopsy-proven grade 3/4 NAFLD fibrosis/cirrhosis (*N* = 14) and age- and sex-matched controls with normal histology (*N* = 15). We identified 208 CpG islands (CGIs), including 99 hypomethylated and 109 hypermethylated CGIs, showing statistically significant evidence (adjusted *P* value < 0.05) for differential methylation between cirrhotic and normal samples. Comparison of *β* values for each CGI to the read count of its corresponding gene obtained from RNA-sequencing analysis revealed negative correlation (adjusted *P* value < 0.05) for 34 transcripts. These findings provide supporting evidence for a role for CpG methylation in the pathogenesis of NAFLD-related cirrhosis, including confirmation of previously reported differentially methylated CGIs, and contribute new insight into the molecular mechanisms underlying the initiation and progression of liver fibrosis and cirrhosis.

**Electronic supplementary material:**

The online version of this article (10.1186/s13148-018-0525-9) contains supplementary material, which is available to authorized users.

## Introduction

Obesity and insulin resistance are commonly associated with fat accumulation in the liver, which is classified as nonalcoholic fatty liver disease (NAFLD) [1, 2]. This common disease encompasses a wide range of liver conditions ranging from hepatic steatosis to nonalcoholic steatohepatitis (NASH) in the absence of significant alcohol consumption [3]. NAFLD/NASH has become a leading cause of cryptogenic cirrhosis [4] and is currently the third leading clinical indication for liver transplantation in the USA [5]. The molecular pathogenesis underlying NAFLD appears to involve many factors, including those of genetic and environmental origins [6].

The relationship between NAFLD and obesity raises the possibility that epigenetic mechanisms associated with “metabolic memory” may be involved [7]. DNA methylation of CpG dinucleotides is a well-characterized epigenetic modification that occurs mostly within CpG islands (CGIs) in promoter regions and functions to regulate gene expression. Evidence supporting a role for DNA methylation in the development of metabolic diseases, including NAFLD, is emerging. For example, diets enriched for energy and certain macronutrients appear to induce transgenerational epigenetic changes in rodent models that predispose offspring to hepatic steatosis and NASH [8]. Changes in DNA methylation have been associated with hepatic steatosis [9, 10] and progression of fibrosis [11], while methyl-depleted diets contribute to the development of steatohepatitis, cirrhosis, and liver cancer in rodents [12, 13]. In humans, hypermethylation of NADH dehydrogenase 6 [14] and PPARGC1A has been correlated with NAFLD [15]. To our knowledge, only a few studies have investigated differential methylation of genes using a genome-wide approach in patients with NASH [16–19]. Several studies have also recently identified changes in the methylation profile of specific genes in NAFLD and NASH [20, 21], and type 2 diabetes (T2D) has been associated with epigenetic modifications in human liver [22].

To complement previous studies, we assessed DNA methylation status in liver biopsies of individuals with histologically documented NAFLD-related cirrhosis in the setting of extreme obesity. Because methylation of CpG sites in promoter regions is related to transcriptional regulation, we also examined the relationship between methylation status and gene expression. We identified over 30 CGIs that were differentially methylated in NAFLD cirrhosis and correlated with hepatic gene expression in the same liver samples. These findings contribute supporting evidence for a role for CpG methylation in the pathogenesis of NAFLD-related cirrhosis, including confirmation of almost 90 previously reported differentially methylated CpG sites, and provide new insight into the molecular mechanisms underlying the initiation and progression of liver fibrosis and cirrhosis.

## Materials and methods

### Study sample

Liver wedge biopsies were intraoperatively obtained from Caucasian women enrolled in the Bariatric Surgery Program at the Geisinger Clinic Center for Nutrition and Weight Management and histologically evaluated using NASH CRN criteria as described [23–25]. Patients with histologic or serologic evidence for other chronic liver diseases were excluded from this study. Both medical history and histological assessment excluded individuals with clinically significant alcohol intake and drug use from participation in the bariatric surgery program. Clinical data, including demographics, clinical measures, ICD-9 codes, medical history, medication use, and common lab results, were available for all study participants as described previously [26]. All study participants provided written informed consent for research, which was conducted according to The Code of Ethics of the World Medical Association (Declaration of Helsinki). The Institutional Review Boards of Geisinger Health System, the Translational Genomics Research Institute, and the Lewis Katz School of Medicine at Temple University approved the research.

### Methylation analysis

Whole genome methylation profiling was performed using the 450K Infinium Methylation BeadChip Assay (Illumina; San Diego, CA, USA). Liver genomic DNA was extracted using the QIAamp DNA kit (Qiagen; Germantown, MD, USA), and DNA concentration was determined using the Quant-iT PicoGreen dsDNA Assay kit (Thermo Fisher Scientific; Waltham, MA). Bisulfite conversion of genomic DNA was performed using the EZ DNA Methylation Kit (Zymo Research; Irvine, CA, USA). Bisulfite-treated DNA was then hybridized to arrays according to the manufacturer’s protocol. Methylation levels for each CpG residue were estimated as the ratio of the methylated signal intensity over the sum of the methylated and unmethylated intensities at each locus using the minfi R package and presented as *β* values [27]. For quality control, we used the publicly available software CpGassoc [28] to exclude any samples with probe detection call rates < 95%, as well as those with an average intensity value of either < 50% of the experiment-wide sample mean or < 2000 arbitrary units (AU). Data points were set to missing with detection *P* values > 0.01. Probes overlapping with copy number variants, as well as those mapping to multiple locations with up to two mismatches, were excluded from the analysis. All samples were checked for atypical raw intensity distributions, and *β* value correlation among others within each respective group. Data were normalized using the SWAN algorithm provided by the minfi package (http://bioconductor.org).

To identify CpG sites showing differentially methylation between fibrotic and normal samples, we used the City of Hope CpG Island Analysis Pipeline (COHCAP) [29] and the minfi package. Differentially methylated site analysis encompasses testing each genomic position for association between methylation and phenotype with a F-test for categorical outcome. CpG sites were defined as methylated if they showed a percentage of methylation > 0.5 and unmethylated if they had *β* values < 0.5. To identify differentially methylated CGI, signal from differentially methylated CpG sites within a CGI was averaged for each sample before group comparison. We used a FDR < 0.05 [30] to define a set of differentially methylated CGIs.

### RNA extraction, sequencing, and analysis

Total RNA was extracted using the RNAeasy kit (Qiagen), quantified using the NanoDrop 2000 spectrophotometer (Thermo Fisher Scientific), and converted to cDNA using the Ovation RNA-Seq System V2 (NuGEN; San Carlos, CA, USA). We performed massively parallel RNA-Seq using polyA-selected RNA from biopsied liver samples using the Illumina HiSeq2000 platform, sequencing to a depth of 60 M 100 bp paired-end reads. During the generation of qseq and fastq files for alignment, low-quality reads were identified and removed, and the indexed reads identified and grouped accordingly. Filtered reads were aligned with the human genome using the Bowtie program [31]. Aligned RNA-Seq reads were imported into the Cufflinks program [32] to assemble alignments into a parsimonious set of transcripts and this set of annotated transcripts were quantified in each sample using DESeq2 [33]. To identify genes with negative expression correlations, we compared *β* values for each CGI to the fold-change of its corresponding gene obtained from RNA-seq analysis. The correlation was estimated using Spearman’s rho statistic, with *P* values computed via the asymptotic *t* distribution approximation.

### Functional enrichment analysis

The Ingenuity Pathway Analysis (IPA) software (Qiagen) was used to identify canonical signaling pathways and network connections associated with CGIs. The significance of the association between CGI and canonical pathway was assessed using two criteria: (1) the ratio of the number of molecules mapped to the pathway and total number of molecules involved in the canonical pathway and (2) the Benjamini-Hochberg corrected *P* value from the right-tailed Fisher exact test.

## Results

### Study sample characteristics

DNA was obtained from liver biopsies from 15 patients with NAFLD, including 4 with stage 3 bridging fibrosis (F3) and 11 with either incomplete cirrhosis or stage 4 (F4) fibrosis (cirrhosis), and 15 individuals with normal liver histology matched by age, sex, BMI at biopsy, and T2D status. The demographic information and clinical characteristics of the study participants are shown in Table 1. Individuals with NAFLD fibrosis had significantly higher serum levels of aspartate aminotransferase (AST), alanine aminotransferase (ALT), insulin, and triglycerides compared to individuals without fibrosis. NAFLD patients with fibrosis also manifested lobular inflammation, portal inflammation, and hepatocyte ballooning.

**Table 1:** Patient demographics and clinical characteristics

	Normal (*N* = 15)	Fibrosis (*N* = 15)	*P* value
Female, *n* (%)	15 (1.00)	15 (1.00)	NS
Mean age in years at biopsy (SD)	47.1 (6.3)	51.3 (8.2)	NS
BMI at biopsy (SD)	41.0 (4.6)	44.4 (6.6)	NS
Diabetes mellitus, *n* (%)	6 (0.40)	6 (0.40)	NS
Laboratory measures, mean (SD)			
Serum AST, U/L	23.2 (4.2)	47.5 (23.3)	.0004
Serum ALT, U/L	23.7 (8.1)	47.3 (26.5)	.0027
AST/ALT, U/L	1.0 (0.3)	1.0 (0.3)	NS
Alkaline phosphatase	71.2 (15.9)	91.6 (80.5)	NS
Total bilirubin	0.49 (0.24)	0.47 (0.18)	NS
Glucose (mg/dL)	100.9 (31.9)	120.2 (53.5)	NS
Insulin	18.0 (13.2)	39.7 (29.8)	.0155
HbA1c, % (SD)	6.0 (1.0)	6.6 (1.3)	NS
Triglycerides	142.9 (64.5)	186.9 (67.2)	0.04
Total cholesterol	179.7 (26.1)	197.9 (33.9)	NS
LDL-C	107.3 (14.4)	116.5 (17.2)	NS
HDL-C	55.9 (28.3)	46.7 (32.4)	NS
Histologic characteristics			
Steatosis, mean grade (SD)	0	2.1 (0.7)	< 0.0001
Lobular inflammation, % = grade 1	0	0.47	< 0.0001
Lobular inflammation, % > grade 2	0	0.4	< 0.0001
Portal inflammation, (%)	0	0.4	< 0.0001
Ballooning (mean score)	0	1.3 (0.7)	< 0.0001

### Identification of differentially methylated CGIs

For each hepatic DNA sample, methylation levels for each CpG residue were estimated as *β* values, which represent the ratio of the methylated signal intensity over the sum of the methylated and unmethylated intensities. Initial hierarchical clustering analysis revealed that F3 fibrosis could be segregated as a separate group from F4, consistent with the distinct histology of stage 3 versus stage 4. To avoid the introduction of unnecessary heterogeneity, we excluded these three samples from further analysis. In addition, one NAFLD fibrosis sample failed quality control measures and was removed from further analysis. Using data from the remaining 11 cirrhosis and 15 normal samples, we identified 208 CGIs, including 99 hypomethylated and 109 hypermethylated CGIs (Additional file 1: Table S1) showing statistically significant evidence (adjusted *P* value < 0.05) for differential methylation. Analysis of individual sites revealed differential methylation of 4275 CpG sites, (adjusted *P* value < 0.05), corresponding to 1713 hypomethylated and 2562 hypermethylated CpG sites; the top 100 differentially methylated CpG sites are shown in Additional file 2: Table S2. The top CGI, prioritized by strength of statistically significant evidence for differential methylation, magnitude of difference in methylation levels (Δ beta), and biological relevance to general liver function or metabolism, are shown in Table 2 (hypermethylation) and Table 3 (hypomethylation).

**Table 2:** CpG islands showing the strongest evidence for hypermethylation in NAFLD cirrhosis

CGI location	Gene ID	Δ Beta	*P* value	FDR
chr5: 127871958-127872263	*FBN2*	0.12	1.29E−06	1.63E−05
chr16: 46917849-46918957	*GPT2*	0.13	4.09E−06	2.95E−05
chr11: 113929633-113932190	*ZBTB16*	0.14	1.33E−05	5.70E−05
chr14: 89882421-89884278	*FOXN3*	0.12	1.39E−05	5.83E−05
chr1: 64058937-64059913	*PGM1*	0.14	1.53E−05	6.32E−05
chr14: 53257664-53258400	*GNPNAT1*	0.16	2.13E−05	7.74E−05
chr8: 134308328-134310145	*NDRG1*	0.15	2.91E−05	9.19E−05
chr21: 47648191-47649622	*LSS*	0.14	3.04E−05	9.39E−05
chr10: 126106818-126107862	*OAT*	0.14	4.24E−05	1.09E−04
chr4: 185724434-185724647	*ACSL1*	0.14	5.77E−05	1.32E−04

**Table 3:** Ten CpG islands showing the strongest evidence for hypomethylation in NAFLD cirrhosis

CGI location	Gene ID	Δ Beta	*P* value	FDR
chr11: 63753414-63754454	*OTUB1*	− 0.14	1.24E−10	2.69E−08
chr1: 155947678-155948490	*ARHGEF2*	− 0.17	1.79E−07	1.09E−05
chr15: 60689749-60690430	*ANXA2*	− 0.15	4.05E−07	1.63E−05
chr8: 59571603-59572526	*NSMAF*	− 0.18	5.93E−07	1.63E−05
chr2: 233925091-233925318	*INPP5D*	− 0.11	7.39E−07	1.63E−05
chr7: 106508057-106508733	*PIK3CG*	− 0.14	8.91E−07	1.63E−05
chr9: 123690771-123691675	*TRAF1*	− 0.16	1.09E−06	1.63E−05
chr15: 72522131-72524238	*PKM2*	− 0.17	8.35E−06	4.44E−05
chr21: 32929927-32932017	*TIAM1*	− 0.17	1.32E−05	5.70E−05
chr11: 128391712-128392611	*ETS1*	− 0.17	1.93E−05	7.38E−05

### Correlation of CpG methylation with hepatic gene expression

To identify gene-methylation correlations, we compared *β* values for each CGI to the FPKM (fragments per kilobase of transcript per million mapped reads) data of its corresponding gene. Focusing on pairwise associations with a significant negative correlation at FDR < 0.05, we found evidence for negative correlation between 34 CGI-transcript pairs (Table 4). Box plots of mean (± standard deviation) methylation *β* values for CGIs associated with gene expression differences in patients with NAFLD fibrosis (*n* = 11) compared to individuals with normal liver histology (*n* = 15) are presented in Additional file 3: Figure S1.

**Table 4:** Integration with gene expression based on correlation statistic

Island	Direction	Gene	Correlation	*P* value	FDR
chr1: 64058937-64059913	Increased methylation	*PGM1*	− 0.69	9.31E−03	3.30E−02
chr1: 6685071-6685691	Increased methylation	*PHF13*	− 0.75	2.92E−03	1.48E−02
chr2: 127817994-127818231	Increased methylation	*BIN1*	− 0.77	2.17E−03	1.16E−02
chr4: 40858884-40859162	Decreased methylation	*APBB2*	− 0.84	3.24E−04	2.60E−03
chr4: 81109887-81110460	Increased methylation	*PRDM8*	− 0.89	4.51E−05	7.26E−04
chr5: 127871958-127872263	Increased methylation	*FBN2*	− 0.88	6.81E−05	1.01E−03
chr6: 13925099-13925510	Increased methylation	*RNF182*	− 0.86	1.39E−04	1.43E−03
chr6: 30038881-30039477	Increased methylation	*RNF39*	− 0.83	4.59E−04	3.53E−03
chr6: 31619856-31620525	Increased methylation	*APOM*	− 0.82	6.27E−04	4.62E−03
chr7: 150417414-150418018	Decreased methylation	*GIMAP1*	− 0.92	9.67E−06	5.82E−04
chr7: 1753446-1753706	Increased methylation	*ELFN1*	− 0.64	1.73E−02	4.94E−02
chr7: 27147589-27148389	Increased methylation	*HOXA3*	− 0.90	2.79E−05	5.82E−04
chr7: 27162087-27162426	Increased methylation	*HOXA3*	− 0.89	4.37E−05	7.26E−04
chr7: 27169572-27170638	Increased methylation	*HOXA4*	− 0.86	1.45E−04	1.43E−03
chr8: 134308328-134310145	Increased methylation	*NDRG1*	− 0.73	4.57E−03	2.07E−02
chr8: 145730390-145732205	Increased methylation	*GPT*	− 0.88	8.23E−05	1.12E−03
chr8: 27348658-27348883	Increased methylation	*EPHX2*	− 0.79	1.20E−03	7.05E−03
chr8: 59571603-59572526	Decreased methylation	*NSMAF*	− 0.79	1.35E−03	7.72E−03
chr9: 99616401-99616940	Decreased methylation	*ZNF782*	− 0.72	5.26E−03	2.27E−02
chr10: 43697777-43698177	Increased methylation	*RASGEF1A*	− 0.90	2.35E−05	5.82E−04
chr11: 118781060-118781732	Decreased methylation	*BCL9L*	− 0.69	8.58E−03	3.10E−02
chr11: 1330390-1331498	Increased methylation	*TOLLIP*	− 0.65	1.69E−02	4.94E−02
chr11: 279072-281700	Increased methylation	*NLRP6*	− 0.71	6.03E−03	2.48E−02
chr11: 70508328-70508617	Decreased methylation	*SHANK2*	− 0.70	7.74E−03	2.98E−02
chr11: 70672834-70673055	Decreased methylation	*SHANK2*	− 0.71	6.72E−03	2.66E−02
chr12: 121163472-121163913	Increased methylation	*ACADS*	− 0.91	1.25E−05	5.82E−04
chr15: 60690708-60690930	Decreased methylation	*ANXA2*	− 0.72	5.51E−03	2.32E−02
chr15: 96864881-96866787	Decreased methylation	*NR2F2*	− 0.78	1.52E−03	8.41E−03
chr17: 8649003-8649513	Increased methylation	*CCDC42*	− 0.91	1.84E−05	5.82E−04
chr18: 56530395-56531288	Decreased methylation	*ZNF532*	− 0.66	1.49E−02	4.70E−02
chr19: 6752515-6753657	Increased methylation	*SH2D3A*	− 0.80	9.31E−04	5.88E−03
chr20:20433105-20433329	Decreased methylation	*RALGAPA2*	− 0.73	4.40E−03	2.06E−02
chr21: 47648191-47649622	Increased methylation	*LSS*	− 0.66	1.35E−02	4.44E−02
chr22: 37420225-37420900	Increased methylation	*MPST*	− 0.81	8.54E−04	5.70E−03

### Functional enrichment analysis

We used functional enrichment analyses to identify the effect of differentially methylated CGI on different canonical pathways. The analysis revealed 113 enriched canonical pathways (Additional file 4: Table S3). Pathways with relevance to NAFLD cirrhosis identified in this analysis are highlighted in Table 5. The top pathways included production of nitric oxide and reactive oxygen species, LXR/RXR activation, and FXR/RXR activation.

**Table 5:** Functional enrichment analysis: canonical pathways

Ingenuity canonical pathways	*P* value	Ratio	Molecules
Production of nitric oxide and reactive oxygen species in macrophages	1.41E−05	0.05	*RHOG*, *APOB*, *PPP1R3C*, *RHOB*, *MAP3K8*, *IRS1*, *RBP4*, *CYBA*, *APOM*, *PIK3CG*
LXR/RXR activation	1.74E−05	0.07	*NCOR2*, *APOB*, *TF*, *VTN*, *RXRA*, *RBP4*, *PON3*, *APOM*
FXR/RXR activation	2.34E−05	0.06	*APOB*, *TF*, *VTN*, *RXRA*, *RBP4*, *NR5A2*, *PON3*, *APOM*
RAR activation	7.24E−05	0.05	*NCOR2*, *ADCY3*, *DUSP1*, *ZBTB16*, *RXRA*, *RBP4*, *ADCY9*, *NR2F2*, *PIK3CG*
B cell receptor signaling	4.27E−04	0.04	*INPP5D*, *PTK2B*, *EBF1*, *MAP3K8*, *IRS1*, *ETS1*, *RASSF5*, *PIK3CG*
Type II diabetes mellitus signaling	1.26E−03	0.05	*ACSL1*, *SLC2A2*, *IRS1*, *PKM*, *NSMAF*, *PIK3CG*
Cardiac hypertrophy signaling	1.66E−03	0.03	*RHOG*, *ADCY3*, *RHOB*, *MAP3K8*, *IRS1*, *CACNA1A*, *ADCY9*, *PIK3CG*
Leptin signaling in obesity	8.51E−03	0.05	*ADCY3*, *IRS1*, *ADCY9*, *PIK3CG*
ERK/MAPK signaling	1.10E−02	0.03	*PPP1R3C*, *DUSP1*, *PTK2B*, *IRS1*, *ETS1*, *PIK3CG*
mTOR signaling	1.12E−02	0.03	*RHOG*, *RPS6*, *RHOB*, *IRS1*, *RPS2*, *PIK3CG*
VEGF signaling	1.55E−02	0.04	*ACTG1*, *PTK2B*, *IRS1*, *PIK3CG*
Maturity onset diabetes of young (MODY) signaling	1.58E−02	0.10	*SLC2A2*, *CACNA1A*
PPARα/RXRα activation	2.63E−02	0.03	*NCOR2*, *ADCY3*, *RXRA*, *IRS1*, *ADCY9*
Atherosclerosis signaling	3.09E−02	0.03	*APOB*, *ITGB2*, *RBP4*, *APOM*
Adipogenesis pathway	3.63E−02	0.03	*PLIN1*, *EBF1*, *NR2F2*, *HDAC7*
B cell development	4.17E−02	0.06	*HLA-DOA*, *HLA-B*
Insulin receptor signaling	4.27E−02	0.03	*INPP5D*, *PPP1R3C*, *IRS1*, *PIK3CG*

## Discussion

To our knowledge, only four studies have investigated methylation profiles in NAFLD patients [16–19]. Although all four studies utilized the same array-based platform for measuring DNA methylation, they differed with respect to analytical strategy, experimental design, and study sample composition. In the first published study, levels of methylation and mRNA expression were assessed in normal-weight controls (*N* = 18) and obese individuals with liver histology consistent with normal (*N* = 18), steatosis (*N* = 12), or NASH (*N* = 15). Seventy-four differentially methylated CpG sites (FDR < 0.004) were found in comparisons of the four phenotypic groups with NAFLD-specific methylation and expression differences observed for nine genes: *ACLY* (ATP citrate lyase), *GALNTL4* (polypeptide N-acetylgalactosaminyltransferase 18), *GRID1* (glutamate ionotropic receptor delta type subunit 1), *IGF1* (insulin like growth factor 1), *IGFBP2* (insulin like growth factor binding protein 2), *IP6K3* (inositol hexakisphosphate kinase 3), *PC* (pyruvate carboxylase), *PLCG1* (phospholipase C gamma 1), and *PRKCE* (protein kinase C epsilon) [16].

In the same year, Murphy et al. [18] identified 69,247 differentially methylated CpG sites, most of which were hypomethylated, in obese patients with advanced (*N* = 23) versus mild (*N* = 33) NAFLD [18]. In that study, methylation was correlated with gene transcript abundance levels for 7% of the differentially methylated CpG sites and methylation at *FGFR2*, *MATA1*, and *CASP1* was validated in a replication cohort. Despite the similar design between that study and the current work, there were notable differences between the two, which could account for the disparate findings. In the published study, (1) the mild fibrosis group was a mix of NAFLD histological types including grade 1 fibrosis; (2) the advanced NAFLD group included stage 3 fibrosis; (3) the BMI range was 32.0–33.8 across case and controls in both discovery and replication cohorts; and (4) the case and control groups were not matched for T2D. More recently, de Mello et al. [17] identified 1292 CpG sites, representing 677 genes that showed differences in DNA methylation between 26 NASH patients and 34 individuals with normal liver histology, independent of T2D status, age, sex, and BMI. In the present work, we also used a homogeneous control group without histopathological manifestations of NAFLD. The lack of a normal control group is widespread in studies of NAFLD, where liver tissue is often obtained in conjunction with clinically indicated biopsies; thus, a high pre-procedure probability of a pathological finding often results in the presence of some level of NAFLD. We also found that stage 3 fibrosis could be segregated as an epigenetically separate group using hierarchical clustering analysis; thus, we removed these samples from further analysis. This level of bioinformatic quality control is critical when analyzing large genomic datasets such as genome-wide methylation array data.

In the most recent study, Hotta et al. [19] identified a number of differentially methylated regions in a comparison of patients with either mild or advanced NAFLD fibrosis from Japan. That study differed from the current study, not only with respect to ethnic background, but also the NAFLD groups were heterogeneous with respect to fibrosis status and neither the mild nor the advanced fibrosis groups were obese [19].

Despite the differences in design, we compared our results with those reported by published studies discussed above and identified 86 common differentially methylated sites (Additional file 5: Table S4). Interestingly, four (*AQP1*, *FGFR2*, *RBP5*, and *MGMT*) overlapped with differentially methylated CpG sites in all four studies, suggesting that these genes may be a common core set with potential importance in disease pathogenesis. *AQP1* (aquaporin 1) encodes a water channel that is overexpressed in fibrosis and cirrhosis and appears to promote hepatic fibrosis through mechanisms involving pathological angiogenesis [34]; association of increased *AQP1* expression is associated with hypomethylation of CpG site found among these studies. *FGFR2* (fibroblast growth factor receptor 2), which was found to be hypomethylated across these studies, has been linked with liver fibrosis. *FGF2* levels were increased in hepatic stellate cell activation [35] and liver cirrhosis [36]. Further, *FGF2* knockout in the carbon tetrachloride mouse model of hepatic injury was associated with decreased collagen expression and protection from liver fibrosis [37], while Brivanib, an inhibitor of FGFR and vascular endothelial growth factor (VEGF) was shown to inhibit activation of hepatic stellate cells in vitro and liver fibrosis in three different animal models [38]. The biological relevance of the other two genes, retinol-binding protein 5 (*RBP5*) and O^6^-methylguanine-DNA methyltransferase (*MGMT*), both of which were hypomethylated in these studies, to the development of NAFLD-related fibrosis remains unknown.

The most highly associated canonical pathways identified through functional enrichment analysis provide additional evidence for epigenetic regulation in the pathophysiology of NAFLD-related cirrhosis. The most highly enriched pathway, Production of Nitric Oxide and Reactive Oxygen Species in Macrophages, implicates a well-studied molecular mechanism in the progression of NAFLD to cirrhosis. In human studies and in rodent models of NAFLD/NASH, increased reactive oxygen species appear to be a central feature of lipotoxicity and inflammation [39]. Although the use of antioxidants as a therapeutic strategy has not resulted in significant effects in either humans or animal models, most studies suffer from one or more apparent weaknesses [40]. Our results suggest that epigenetic modulation of antioxidant pathways could be a promising therapeutic approach. The next two associated pathways, LXR/RXR activation and FXR/RXR activation, are mechanistically linked via heterodimerization of the nuclear receptors farnesoid X receptor (FXR) and retinoid X receptor (RXR) [41]. FXR is highly expressed in the liver and is involved in several key metabolic processes including bile acid synthesis, glucose and lipid metabolism, and regulation of inflammatory pathways [42]. Administration of the synthetic bile acid derivative and FXR agonist, obeticholic acid, has been shown to reduce the histological NAFLD fibrosis score in a multicenter, randomized, double blind, placebo-controlled study [43]. To date, the role of epigenetic regulation of this pathway in the potential clinical effectiveness of bile acid-based therapeutics has not yet been explored.

Despite the overlap in findings of differential methylation among studies, our sample size, while comparable to several other reports [16, 44, 45], is still limited. We dichotomized our cohort into extreme histologic phenotypes to increase our power to identify clinically relevant differences in DNA methylation levels and were therefore unable to stratify into phenotypic groups to assess intermediate levels of peri-sinusoidal fibrosis or portal fibrosis. Additional studies including individuals spanning the spectrum of NAFLD fibrosis will be critical to extend our findings. We also note that the cross-sectional design does not allow associations with disease progression to be drawn. In the absence of serial liver biopsies in the present cohort, we were not able to utilize a prospective design. Assessment of methylation levels in longitudinal biopsies will be necessary to determine the roles of specific candidates in disease progression. In addition, due to the small sample size in this study, we focused our investigation on females with extreme obesity, who underwent gastric bypass surgery and had liver biopsies. We utilized this design because obesity is a significant risk factor for NAFLD [46], and liver biopsy tissue was obtained without clinical indication, which effectively eliminates bias toward clinically suspect liver disease. Despite the relative phenotypic homogeneity, we were able to replicate findings of differential methylation from study samples encompassing a greater range of phenotypic diversity, indicating the influence of shared epigenetic effects. However, conclusions with regard to NAFLD-related fibrosis obtained from this cohort may still not be relevant to populations with less severe obesity or of different ethnicities without additional validation.

In summary, the results obtained in the current study not only confirm previous findings of differential methylation in NAFLD patients but also provide novel evidence of differential methylation and RNA expression profiles associated with NAFLD-related fibrosis. Future studies will be needed to determine the extent to which DNA methylation patterns in the liver are represented in other metabolically relevant tissues such as visceral and subcutaneous fat [47, 48], as well as peripheral blood leukocytes, which will be critical for the development of non-invasive markers of NAFLD stage. Additional studies, including those showing functional consequences of differentially methylated sites, i.e., disruption of transcription factor binding, will be necessary to confirm the role of specific CpG loci in liver fibrosis. These approaches are expected to yield new insights into the pathological mechanisms underlying the development of fibrosis and cirrhosis in NAFLD.

## Additional files

Additional file 1: **Table S1.** CpG islands differentially methylated between normal and fibrotic samples (*N* = 208) (DOCX 37 kb)

Additional file 2: **Table S2.** Top 100 differentially methylated CpG sites (DOCX 30 kb)

Additional file 3: **Figure S1.** Box plot of mean (± standard deviation) methylation *β* values for CGI associated with gene expression differences in patients with NAFLD fibrosis (*n* = 11) compared to individuals with normal liver histology (*n* = 15). (PDF 544 kb)

Additional file 4: **Table S3.** Canonical pathways identified by pathway analysis using all significant CpG islands (*N* = 208). (DOCX 26 kb)

Additional file 5: **Table S4.** Validation of previously reported methylation findings. (DOCX 34 kb)

## Abbreviations

*ACLY*: ATP citrate lyase
ALT: Alanine aminotransaminase
AQP1: Aquaporin 1
AST: Aspartate aminotransaminase
BMI: Body mass index
CASP1: Caspase 1
CGIs: Cytosine phosphate guanine islands
COHCAP: City of Hope CpG Island Analysis Pipeline
CpG: Cytosine phosphate guanine
FDR: False discovery rate
FGFR2: Fibroblast growth factor receptor 2
FPKM: Fragments per kilobase of transcript per million mapped reads
FXR: Farnesoid X receptor
*GALNTL4*: Polypeptide *N*-acetylgalactosaminyltransferase 18
*GRID1*: Glutamate ionotropic receptor delta type subunit 1
ICD-9: International Classification of Diseases-ninth revision
*IGF1*: Insulin like growth factor 1
*IGFBP2*: Insulin-like growth factor binding protein 2
*IP6K3*: Inositol hexakisphosphate kinase 3
IPA: Ingenuity Pathway Analysis
LXR: Liver X receptor
MATA1: Methionine adenosyltransferase 1A
MGMT: O^6^-Methylguanine-DNA methyltransferase
NADH: Nicotinamide adenine dinucleotide
NAFLD: Nonalcoholic fatty liver disease
NASH CRN: Nonalcoholic steatohepatitis clinical research network
NASH: Nonalcoholic steatohepatitis
*PC*: Pyruvate carboxylase
*PLCG1*: Phospholipase C gamma 1
PPARGC1A: Peroxisome proliferator-activated receptor gamma coactivator 1-alpha
*PRKCE*: Protein kinase C epsilon
RBP5: Retinol binding protein 5
RXR: Retinoid X receptor
T2D: Type 2 diabetes
VEGF: Vascular endothelial growth factor

## References

[1] Wang Y, Li YY, Nie YQ, Zhou YJ, Cao CY, Xu L (2013). Association between metabolic syndrome and the development of non-alcoholic fatty liver disease. Exp Ther Med.

[2] Asrih M, Jornayvaz FR (2015). Metabolic syndrome and nonalcoholic fatty liver disease: is insulin resistance the link?. Mol Cell Endocrinol.

[3] Neuschwander-Tetri BA, Caldwell SH (2003). Nonalcoholic steatohepatitis: summary of an AASLD Single Topic Conference. Hepatology.

[4] Starley BQ, Calcagno CJ, Harrison SA (2010). Nonalcoholic fatty liver disease and hepatocellular carcinoma: a weighty connection. Hepatology.

[5] Wong RJ, Cheung R, Ahmed A (2014). Nonalcoholic steatohepatitis is the most rapidly growing indication for liver transplantation in patients with hepatocellular carcinoma in the US. Hepatology.

[6] Oliveira CP, Stefano JT (2015). Genetic polymorphisms and oxidative stress in non-alcoholic steatohepatitis (NASH): a mini review. Clin Res Hepatol Gastroenterol.

[7] Cencioni C, Spallotta F, Greco S, Martelli F, Zeiher AM, Gaetano C (2014). Epigenetic mechanisms of hyperglycemic memory. Int J Biochem Cell Biol.

[8] Pruis MG, Lendvai A, Bloks VW, Zwier MV, Baller JF, de Bruin A, Groen AK, Plosch T (2014). Maternal western diet primes non-alcoholic fatty liver disease in adult mouse offspring. Acta Physiol (Oxf).

[9] Chang X, Yan H, Fei J, Jiang M, Zhu H, Lu D, Gao X (2010). Berberine reduces methylation of the MTTP promoter and alleviates fatty liver induced by a high-fat diet in rats. J Lipid Res.

[10] Cordero P, Gomez-Uriz AM, Campion J, Milagro FI, Martinez JA. Dietary supplementation with methyl donors reduces fatty liver and modifies the fatty acid synthase DNA methylation profile in rats fed an obesogenic diet. Genes Nutr. 2012; 10.1007/s12263-012-0300-z PMC3534997 22648174

[11] Tian W, Xu H, Fang F, Chen Q, Xu Y, Shen A (2013). Brahma-related gene 1 bridges epigenetic regulation of proinflammatory cytokine production to steatohepatitis in mice. Hepatology.

[12] Lu SC, Alvarez L, Huang ZZ, Chen L, An W, Corrales FJ, Avila MA, Kanel G, Mato JM (2001). Methionine adenosyltransferase 1A knockout mice are predisposed to liver injury and exhibit increased expression of genes involved in proliferation. Proc Natl Acad Sci U S A.

[13] Martinez-Chantar ML, Corrales FJ, Martinez-Cruz LA, Garcia-Trevijano ER, Huang ZZ, Chen L, Kanel G, Avila MA, Mato JM, Lu SC (2002). Spontaneous oxidative stress and liver tumors in mice lacking methionine adenosyltransferase 1A. FASEB J.

[14] Pirola CJ, Fernandez Gianotti T, Burgueno AL, Rey-Funes M, Loidl CF, Mallardi P, San Martino J, Castano GO, Sookoian S. Epigenetic modification of liver mitochondrial DNA is associated with histological severity of nonalcoholic fatty liver disease. Gut. 2012; 10.1136/gutjnl-2012-302962 22879518

[15] Sookoian S, Rosselli MS, Gemma C, Burgueno AL, Fernandez Gianotti T, Castano GO, Pirola CJ (2010). Epigenetic regulation of insulin resistance in nonalcoholic fatty liver disease: impact of liver methylation of the peroxisome proliferator-activated receptor gamma coactivator 1alpha promoter. Hepatology.

[16] Ahrens M, Ammerpohl O, von Schonfels W, Kolarova J, Bens S, Itzel T, Teufel A, Herrmann A, Brosch M, Hinrichsen H (2013). DNA methylation analysis in nonalcoholic fatty liver disease suggests distinct disease-specific and remodeling signatures after bariatric surgery. Cell Metab.

[17] de Mello VD, Matte A, Perfilyev A, Mannisto V, Ronn T, Nilsson E, Kakela P, Ling C, Pihlajamaki J (2017). Human liver epigenetic alterations in non-alcoholic steatohepatitis are related to insulin action. Epigenetics.

[18] Murphy SK, Yang H, Moylan CA, Pang H, Dellinger A, Abdelmalek MF, Garrett ME, Ashley-Koch A, Suzuki A, Tillmann HL (2013). Relationship between methylome and transcriptome in patients with nonalcoholic fatty liver disease. Gastroenterology.

[19] Hotta K, Kitamoto T, Kitamoto A, Ogawa Y, Honda Y, Kessoku T, Yoneda M, Imajo K, Tomeno W, Saito S, Nakajima A. Identification of the genomic region under epigenetic regulation during non-alcoholic fatty liver disease progression. Hepatol Res. 2017; 10.1111/hepr.12992 29059699

[20] Zeybel M, Hardy T, Robinson SM, Fox C, Anstee QM, Ness T, Masson S, Mathers JC, French J, White S, Mann J (2015). Differential DNA methylation of genes involved in fibrosis progression in non-alcoholic fatty liver disease and alcoholic liver disease. Clin Epigenetics.

[21] Kitamoto T, Kitamoto A, Ogawa Y, Honda Y, Imajo K, Saito S, Yoneda M, Nakamura T, Nakajima A, Hotta K (2015). Targeted-bisulfite sequence analysis of the methylation of CpG islands in genes encoding PNPLA3, SAMM50, and PARVB of patients with non-alcoholic fatty liver disease. J Hepatol.

[22] Nilsson E, Matte A, Perfilyev A, de Mello VD, Kakela P, Pihlajamaki J, Ling C (2015). Epigenetic alterations in human liver from subjects with type 2 diabetes in parallel with reduced folate levels. J Clin Endocrinol Metab.

[23] Leti F, Malenica I, Doshi M, Courtright A, Van Keuren-Jensen K, Legendre C, Still CD, Gerhard GS, DiStefano JK. High-throughput sequencing reveals altered expression of hepatic microRNAs in nonalcoholic fatty liver disease-related fibrosis. Transl Res. 2015; 10.1016/j.trsl.2015.04.014 PMC4537840 26001595

[24] DiStefano JK, Kingsley C, Craig Wood G, Chu X, Argyropoulos G, Still CD, Done SC, Legendre C, Tembe W, Gerhard GS (2015). Genome-wide analysis of hepatic lipid content in extreme obesity. Acta Diabetol.

[25] Gerhard GS, Benotti P, Wood GC, Chu X, Argyropoulos G, Petrick A, Strodel WE, Gabrielsen JD, Ibele A, Still CD (2014). Identification of novel clinical factors associated with hepatic fat accumulation in extreme obesity. J Obes.

[26] Wood GC, Chu X, Manney C, Strodel W, Petrick A, Gabrielsen J, Seiler J, Carey D, Argyropoulos G, Benotti P (2012). An electronic health record-enabled obesity database. BMC Med Inform Decis Mak.

[27] Network TCGAR (2008). Comprehensive genomic characterization defines human glioblastoma genes and core pathways. Nature.

[28] Barfield RT, Kilaru V, Smith AK, Conneely KN (2012). CpGassoc: an R function for analysis of DNA methylation microarray data. Bioinformatics.

[29] Warden CD, Lee H, Tompkins JD, Li X, Wang C, Riggs AD, Yu H, Jove R, Yuan YC (2013). COHCAP: an integrative genomic pipeline for single-nucleotide resolution DNA methylation analysis. Nucleic Acids Res.

[30] Storey JD (2002). A direct approach to false discovery rates. Journal of the Royal Statistical Society: Series B (Statistical Methodology).

[31] Langmead B, Trapnell C, Pop M, Salzberg SL (2009). Ultrafast and memory-efficient alignment of short DNA sequences to the human genome. Genome Biol.

[32] Trapnell C, Williams BA, Pertea G, Mortazavi A, Kwan G, van Baren MJ, Salzberg SL, Wold BJ, Pachter L (2010). Transcript assembly and quantification by RNA-Seq reveals unannotated transcripts and isoform switching during cell differentiation. Nat Biotechnol.

[33] Anders S, Huber W (2010). Differential expression analysis for sequence count data. Genome Biol.

[34] Huebert RC, Vasdev MM, Shergill U, Das A, Huang BQ, Charlton MR, LaRusso NF, Shah VH (2010). Aquaporin-1 facilitates angiogenic invasion in the pathological neovasculature that accompanies cirrhosis. Hepatology.

[35] Fibbi G, Pucci M, Grappone C, Pellegrini G, Salzano R, Casini A, Milani S, Del Rosso M (1999). Functions of the fibrinolytic system in human Ito cells and its control by basic fibroblast and platelet-derived growth factor. Hepatology.

[36] Jin-no K, Tanimizu M, Hyodo I, Kurimoto F, Yamashita T (1997). Plasma level of basic fibroblast growth factor increases with progression of chronic liver disease. J Gastroenterol.

[37] Yu C, Wang F, Jin C, Huang X, Miller DL, Basilico C, McKeehan WL (2003). Role of fibroblast growth factor type 1 and 2 in carbon tetrachloride-induced hepatic injury and fibrogenesis. Am J Pathol.

[38] Nakamura I, Zakharia K, Banini BA, Mikhail DS, Kim TH, Yang JD, Moser CD, Shaleh HM, Thornburgh SR, Walters I, Roberts LR (2014). Brivanib attenuates hepatic fibrosis in vivo and stellate cell activation in vitro by inhibition of FGF, VEGF and PDGF signaling. PLoS One.

[39] Sunny NE, Bril F, Cusi K (2017). Mitochondrial adaptation in nonalcoholic fatty liver disease: novel mechanisms and treatment strategies. Trends Endocrinol Metab.

[40] Torok NJ (2016). Dysregulation of redox pathways in liver fibrosis. Am J Physiol Gastrointest Liver Physiol.

[41] Rizzo G, Renga B, Mencarelli A, Pellicciari R, Fiorucci S (2005). Role of FXR in regulating bile acid homeostasis and relevance for human diseases. Curr Drug Targets Immune Endocr Metabol Disord.

[42] Armstrong LE, Guo GL (2017). Role of FXR in liver inflammation during nonalcoholic steatohepatitis. Curr Pharmacol Rep.

[43] Oseini AM, Sanyal AJ (2017). Therapies in non-alcoholic steatohepatitis (NASH). Liver Int.

[44] Cheung O, Puri P, Eicken C, Contos MJ, Mirshahi F, Maher JW, Kellum JM, Min H, Luketic VA, Sanyal AJ (2008). Nonalcoholic steatohepatitis is associated with altered hepatic microRNA expression. Hepatology.

[45] Estep M, Armistead D, Hossain N, Elarainy H, Goodman Z, Baranova A, Chandhoke V, Younossi ZM (2010). Differential expression of miRNAs in the visceral adipose tissue of patients with non-alcoholic fatty liver disease. Aliment Pharmacol Ther.

[46] Chiang DJ, Pritchard MT, Nagy LE (2011). Obesity, diabetes mellitus, and liver fibrosis. Am J Physiol Gastrointest Liver Physiol.

[47] Duval C, Thissen U, Keshtkar S, Accart B, Stienstra R, Boekschoten MV, Roskams T, Kersten S, Muller M (2010). Adipose tissue dysfunction signals progression of hepatic steatosis towards nonalcoholic steatohepatitis in C57BL/6 mice. Diabetes.

[48] Lomonaco R, Ortiz-Lopez C, Orsak B, Webb A, Hardies J, Darland C, Finch J, Gastaldelli A, Harrison S, Tio F, Cusi K (2012). Effect of adipose tissue insulin resistance on metabolic parameters and liver histology in obese patients with nonalcoholic fatty liver disease. Hepatology.

