# Supplementary material for: Differentially methylated loci in NAFLD cirrhosis are associated with key signaling pathways
Source: Clin Epigenetics. 2018 Jul 13;10:93. doi: 10.1186/s13148-018-0525-9 (PMC6044005; doi:10.1186/s13148-018-0525-9)
Supplement: Supplementary file 1 — Table S1. CpG islands differentially methylated between normal and fibrotic samples (N = 208) (DOCX 37 kb) [file 13148_2018_525_MOESM1_ESM.docx]

|  | | | | |
| --- | --- | --- | --- | --- |
| **Table S1. CpG islands differentially methylated between normal and fibrotic samples (N=208)** | | | | |
| **Island location** | **Gene ID** | **Δ Beta** | **FDR** | **#CpG Sites** |
| chr11:63753414-63754454 | OTUB1 | -0.14 | 2.69E-08 | 3 |
| chr6:13925099-13925510 | RNF182 | 0.13 | 1.00E-05 | 2 |
| chr1:155947678-155948490 | ARHGEF2 | -0.17 | 1.10E-05 | 4 |
| chr6:31509668-31509896 | BAT1 | -0.12 | 1.10E-05 | 2 |
| chr5:127871958-127872263 | FBN2 | 0.12 | 1.63E-05 | 2 |
| chr11:2923301-2923817 | SLC22A18;SLC22A18AS | -0.12 | 1.63E-05 | 2 |
| chr11:44586602-44587404 | CD82 | -0.15 | 1.63E-05 | 2 |
| chr11:63974829-63975048 | FERMT3 | -0.11 | 1.63E-05 | 5 |
| chr12:123380333-123380894 | VPS37B | -0.14 | 1.63E-05 | 2 |
| chr15:60689749-60690430 | ANXA2 | -0.15 | 1.63E-05 | 2 |
| chr19:17000627-17001398 | F2RL3 | -0.14 | 1.63E-05 | 2 |
| chr2:233925091-233925318 | INPP5D | -0.11 | 1.63E-05 | 2 |
| chr20:20433105-20433329 | RALGAPA2 | -0.12 | 1.63E-05 | 2 |
| chr21:46351328-46352911 | ITGB2 | -0.14 | 1.63E-05 | 3 |
| chr7:100183527-100184625 | LRCH4 | -0.14 | 1.63E-05 | 2 |
| chr7:106508057-106508733 | PIK3CG | -0.14 | 1.63E-05 | 5 |
| chr8:59571603-59572526 | NSMAF | -0.18 | 1.63E-05 | 2 |
| chr9:123690771-123691675 | TRAF1 | -0.16 | 1.63E-05 | 2 |
| chr4:81109887-81110460 | PRDM8 | 0.12 | 2.06E-05 | 2 |
| chr19:14551998-14552255 | PKN1 | -0.12 | 2.06E-05 | 2 |
| chr19:35491151-35492020 | GRAMD1A | -0.13 | 2.52E-05 | 2 |
| chr4:2819499-2820429 | SH3BP2 | -0.14 | 2.52E-05 | 2 |
| chr8:145024449-145025179 | PLEC1 | -0.11 | 2.52E-05 | 2 |
| chr2:85980499-85982198 | ATOH8 | -0.12 | 2.55E-05 | 2 |
| chr16:46917849-46918957 | GPT2 | 0.13 | 2.96E-05 | 2 |
| chr20:36148603-36150136 | NNAT;BLCAP | 0.15 | 2.96E-05 | 2 |
| chr10:103879633-103880628 | LDB1 | -0.14 | 2.96E-05 | 3 |
| chr10:120966127-120967623 | GRK5 | -0.12 | 2.96E-05 | 3 |
| chr11:118781060-118781732 | BCL9L | -0.13 | 2.96E-05 | 2 |
| chr11:3861855-3862996 | RHOG | -0.13 | 2.96E-05 | 2 |
| chr11:70672834-70673055 | SHANK2 | -0.12 | 2.96E-05 | 3 |
| chr12:6649677-6649897 | IFFO1 | -0.11 | 2.96E-05 | 2 |
| chr6:43252734-43253476 | TTBK1 | -0.11 | 3.16E-05 | 3 |
| chr3:39186524-39186730 | CSRNP1 | -0.15 | 3.19E-05 | 2 |
| chr11:86748660-86749093 | TMEM135 | 0.15 | 3.39E-05 | 2 |
| chr17:40937258-40937480 | WNK4 | -0.13 | 3.71E-05 | 2 |
| chr4:3374733-3374998 | RGS12 | -0.12 | 3.71E-05 | 2 |
| chr12:3862068-3862606 | EFCAB4B | -0.15 | 4.09E-05 | 4 |
| chr1:1370767-1371449 | VWA1 | -0.12 | 4.43E-05 | 3 |
| chr7:150417414-150418018 | GIMAP1 | -0.11 | 4.43E-05 | 3 |
| chr15:72522131-72524238 | PKM2 | -0.17 | 4.44E-05 | 2 |
| chr19:36134153-36135781 | ETV2 | -0.13 | 4.57E-05 | 2 |
| chr17:81009245-81009999 | B3GNTL1 | -0.13 | 4.96E-05 | 2 |
| chr5:158523906-158524598 | EBF1 | 0.12 | 5.01E-05 | 3 |
| chr12:54071053-54071265 | ATP5G2 | -0.10 | 5.01E-05 | 2 |
| chr9:19379794-19380341 | RPS6 | -0.11 | 5.01E-05 | 2 |
| chr19:47290585-47291983 | SLC1A5 | -0.15 | 5.28E-05 | 4 |
| chr19:13944139-13944695 | MIR27A;MIR23A;MIR24-2 | -0.12 | 5.56E-05 | 3 |
| chr15:37390175-37390380 | MEIS2 | -0.13 | 5.56E-05 | 3 |
| chr11:113929633-113932190 | ZBTB16 | 0.14 | 5.70E-05 | 2 |
| chr21:32929927-32932017 | TIAM1 | -0.17 | 5.70E-05 | 2 |
| chr14:89882421-89884278 | FOXN3 | 0.12 | 5.83E-05 | 2 |
| chr1:64058937-64059913 | PGM1 | 0.14 | 6.32E-05 | 2 |
| chr1:206730397-206730908 | RASSF5 | -0.12 | 6.45E-05 | 2 |
| chr8:145730390-145732205 | GPT | 0.11 | 7.38E-05 | 2 |
| chr11:128391712-128392611 | ETS1 | -0.17 | 7.38E-05 | 2 |
| chr9:132597219-132598321 | C9orf78;USP20 | -0.15 | 7.38E-05 | 2 |
| chr14:53257664-53258400 | GNPNAT1 | 0.16 | 7.74E-05 | 2 |
| chr3:190039817-190040465 | CLDN1 | 0.11 | 7.74E-05 | 2 |
| chr7:100075303-100075551 | TSC22D4 | -0.11 | 7.74E-05 | 3 |
| chr14:103593234-103593923 | TNFAIP2 | 0.11 | 7.86E-05 | 2 |
| chr1:150121695-150123078 | PLEKHO1 | -0.12 | 7.86E-05 | 2 |
| chr8:27183090-27183390 | PTK2B | -0.13 | 7.86E-05 | 2 |
| chr10:30722378-30723707 | MAP3K8 | -0.11 | 7.89E-05 | 2 |
| chr10:102279162-102279730 | SEC31B | 0.13 | 8.47E-05 | 3 |
| chr11:67350928-67351953 | GSTP1 | -0.13 | 8.85E-05 | 2 |
| chr16:4015001-4015232 | ADCY9 | -0.16 | 9.03E-05 | 2 |
| chr8:134308328-134310145 | NDRG1 | 0.15 | 9.19E-05 | 2 |
| chr15:90208787-90209279 | PLIN1 | -0.11 | 9.19E-05 | 2 |
| chr21:47648191-47649622 | LSS | 0.14 | 9.39E-05 | 2 |
| chr6:7141036-7141576 | RREB1 | 0.15 | 9.39E-05 | 3 |
| chr12:48206721-48207126 | HDAC7 | -0.12 | 9.39E-05 | 2 |
| chr17:79478235-79482426 | ACTG1 | -0.17 | 9.39E-05 | 4 |
| chr17:33776553-33776888 | SLFN13 | 0.11 | 9.43E-05 | 2 |
| chr16:88716989-88717606 | CYBA | -0.13 | 9.43E-05 | 6 |
| chr12:124941314-124941584 | NCOR2 | -0.12 | 9.77E-05 | 4 |
| chr15:65822685-65823540 | PTPLAD1 | 0.11 | 1.02E-04 | 2 |
| chr8:22224519-22225244 | SLC39A14 | 0.13 | 1.07E-04 | 2 |
| chr20:25037707-25039211 | ACSS1 | -0.14 | 1.07E-04 | 3 |
| chr11:47736739-47737106 | AGBL2 | 0.13 | 1.09E-04 | 2 |
| chr17:8649003-8649513 | CCDC42 | 0.12 | 1.09E-04 | 2 |
| chr1:200004474-200004933 | NR5A2 | -0.12 | 1.09E-04 | 4 |
| chr11:70508328-70508617 | SHANK2 | -0.12 | 1.09E-04 | 5 |
| chr10:126106818-126107862 | OAT | 0.14 | 1.09E-04 | 2 |
| chr3:156533839-156535131 | PA2G4P4 | 0.13 | 1.09E-04 | 2 |
| chr10:13344235-13344498 | PHYH | 0.11 | 1.10E-04 | 2 |
| chr9:130699759-130700266 | DPM2 | 0.13 | 1.10E-04 | 2 |
| chr10:3823789-3824017 | KLF6 | -0.11 | 1.10E-04 | 2 |
| chr19:50037078-50037670 | RCN3 | -0.13 | 1.17E-04 | 2 |
| chr1:55352463-55353328 | DHCR24 | 0.11 | 1.18E-04 | 2 |
| chr11:118401235-118402069 | TTC36 | 0.13 | 1.20E-04 | 3 |
| chr6:160511936-160512465 | IGF2R;LOC729603 | 0.13 | 1.23E-04 | 2 |
| chr19:10172698-10172900 | C3P1 | -0.13 | 1.23E-04 | 3 |
| chr4:169930790-169931607 | CBR4 | 0.12 | 1.31E-04 | 2 |
| chr4:185724434-185724647 | ACSL1 | 0.14 | 1.32E-04 | 5 |
| chr12:54784900-54785238 | ZNF385A | -0.15 | 1.35E-04 | 2 |
| chr7:27162087-27162426 | HOXA3 | 0.12 | 1.39E-04 | 3 |
| chr8:55047705-55048005 | MRPL15 | 0.10 | 1.39E-04 | 2 |
| chr17:66596072-66597578 | FAM20A | 0.14 | 1.42E-04 | 2 |
| chr7:24796541-24797487 | DFNA5 | 0.15 | 1.42E-04 | 3 |
| chr5:141348531-141349104 | RNF14 | 0.14 | 1.49E-04 | 2 |
| chr17:79008929-79009762 | BAIAP2 | 0.15 | 1.50E-04 | 6 |
| chr19:4909262-4910256 | UHRF1 | -0.13 | 1.50E-04 | 2 |
| chr17:3375006-3375237 | ASPA | 0.13 | 1.56E-04 | 2 |
| chr20:62374061-62374353 | SLC2A4RG | 0.18 | 1.56E-04 | 2 |
| chr7:95025559-95026122 | PON3 | 0.12 | 1.57E-04 | 3 |
| chr10:93392667-93393147 | PPP1R3C | 0.12 | 1.59E-04 | 2 |
| chr3:170746072-170746292 | SLC2A2 | 0.11 | 1.59E-04 | 2 |
| chr7:1753446-1753706 | ELFN1 | 0.13 | 1.63E-04 | 2 |
| chr12:121163472-121163913 | ACADS | 0.15 | 1.65E-04 | 2 |
| chr5:172197482-172199606 | DUSP1 | 0.13 | 1.68E-04 | 2 |
| chr2:25142472-25143689 | ADCY3 | -0.14 | 1.84E-04 | 2 |
| chr14:24779874-24780932 | CIDEB;LTB4R2 | 0.11 | 1.87E-04 | 2 |
| chr22:24890412-24891453 | C22orf45;UPB1 | 0.13 | 1.87E-04 | 4 |
| chr13:101184396-101184848 | A2LD1 | 0.11 | 1.97E-04 | 2 |
| chr10:43697777-43698177 | RASGEF1A | 0.12 | 2.02E-04 | 2 |
| chr17:18266422-18267007 | SHMT1 | 0.13 | 2.02E-04 | 2 |
| chr7:27147589-27148389 | HOXA3 | 0.11 | 2.02E-04 | 2 |
| chr2:10219802-10221016 | CYS1 | -0.13 | 2.02E-04 | 2 |
| chr11:1330390-1331498 | TOLLIP | 0.12 | 2.06E-04 | 2 |
| chr14:100795072-100795376 | C14orf68 | 0.11 | 2.06E-04 | 2 |
| chr21:45875391-45877326 | LRRC3 | 0.13 | 2.06E-04 | 3 |
| chr12:56122017-56123186 | CD63 | -0.10 | 2.07E-04 | 2 |
| chr11:1315467-1317115 | TOLLIP | 0.13 | 2.15E-04 | 2 |
| chr16:4730262-4730487 | MGRN1 | 0.14 | 2.15E-04 | 2 |
| chr10:45496275-45496550 | C10orf25;ZNF22 | 0.12 | 2.16E-04 | 2 |
| chr17:26698359-26699557 | VTN;SEBOX | 0.10 | 2.16E-04 | 2 |
| chr20:62185073-62185572 | C20orf195 | -0.12 | 2.16E-04 | 2 |
| chr10:135341255-135342561 | CYP2E1 | 0.12 | 2.20E-04 | 4 |
| chr17:17625605-17628500 | RAI1 | -0.12 | 2.20E-04 | 2 |
| chr1:220101132-220102147 | SLC30A10 | 0.13 | 2.24E-04 | 2 |
| chr15:96864881-96866787 | NR2F2 | -0.13 | 2.25E-04 | 3 |
| chr11:57267006-57267316 | SLC43A1 | 0.11 | 2.25E-04 | 3 |
| chr7:41745178-41745462 | LOC285954 | 0.15 | 2.25E-04 | 2 |
| chr9:99616401-99616940 | ZNF782 | -0.11 | 2.25E-04 | 2 |
| chr4:1164515-1166582 | SPON2;LOC100130872-SPON2 | 0.11 | 2.31E-04 | 2 |
| chr9:137229726-137229931 | RXRA | 0.13 | 2.31E-04 | 2 |
| chr12:52115410-52115679 | SCN8A | -0.13 | 2.31E-04 | 2 |
| chr18:56530395-56531288 | ZNF532 | -0.11 | 2.31E-04 | 2 |
| chr2:66660452-66660794 | MEIS1 | -0.15 | 2.31E-04 | 2 |
| chr16:2088753-2088998 | SLC9A3R2 | 0.13 | 2.36E-04 | 2 |
| chr2:20646428-20647988 | RHOB | 0.12 | 2.57E-04 | 2 |
| chr5:43042107-43043477 | LOC153684 | 0.16 | 2.57E-04 | 3 |
| chr5:137610105-137610311 | GFRA3 | -0.15 | 2.57E-04 | 3 |
| chr7:142985047-142985810 | TMEM139 | 0.10 | 2.58E-04 | 2 |
| chr2:128421719-128422182 | LIMS2 | 0.13 | 2.62E-04 | 4 |
| chr2:21266669-21266961 | APOB | 0.10 | 2.62E-04 | 2 |
| chr5:1108802-1109051 | SLC12A7 | 0.11 | 2.62E-04 | 2 |
| chr18:55103154-55108853 | ONECUT2 | -0.11 | 2.62E-04 | 2 |
| chr19:54369387-54369809 | MYADM | -0.19 | 2.66E-04 | 2 |
| chr17:46685244-46685449 | HOXB6;LOC404266 | 0.10 | 2.67E-04 | 2 |
| chr6:30640431-30640853 | KIAA1949 | 0.11 | 2.92E-04 | 4 |
| chr20:37075227-37075854 | SNHG11;SNORA39;SNORA60 | 0.14 | 3.07E-04 | 2 |
| chr1:1691767-1692063 | NADK | 0.13 | 3.17E-04 | 5 |
| chr6:32975684-32975926 | HLA-DOA | 0.11 | 3.17E-04 | 3 |
| chr7:27169572-27170638 | HOXA4 | 0.13 | 3.17E-04 | 2 |
| chr8:27348658-27348883 | EPHX2 | 0.12 | 3.17E-04 | 2 |
| chr5:1099394-1099736 | SLC12A7 | 0.15 | 3.17E-04 | 3 |
| chr17:4692249-4693977 | GLTPD2 | 0.12 | 3.17E-04 | 2 |
| chr1:226186803-226187336 | C1orf55 | -0.17 | 3.17E-04 | 3 |
| chr19:4326878-4327380 | STAP2 | -0.13 | 3.18E-04 | 2 |
| chr9:124461797-124462190 | DAB2IP | -0.11 | 3.18E-04 | 2 |
| chr6:31619856-31620525 | APOM | 0.11 | 3.19E-04 | 5 |
| chr8:63998227-63998737 | TTPA | 0.13 | 3.40E-04 | 2 |
| chr1:41249090-41250161 | KCNQ4 | -0.13 | 3.40E-04 | 2 |
| chr2:171569877-171573904 | SP5 | -0.12 | 3.40E-04 | 2 |
| chr11:910677-911581 | CHID1 | 0.12 | 3.40E-04 | 2 |
| chr3:133464949-133465420 | TF | 0.12 | 3.40E-04 | 2 |
| chr13:20766208-20767779 | GJB2 | -0.13 | 3.40E-04 | 2 |
| chr3:11034446-11035384 | SLC6A1 | 0.12 | 3.45E-04 | 2 |
| chr10:95360389-95361387 | RBP4 | 0.14 | 3.47E-04 | 2 |
| chr17:76836060-76837438 | USP36 | 0.11 | 3.47E-04 | 2 |
| chr6:150070513-150071285 | PCMT1 | 0.12 | 3.91E-04 | 2 |
| chr16:30456730-30457071 | SEPHS2 | 0.15 | 3.98E-04 | 2 |
| chr19:11450016-11450414 | RAB3D | -0.15 | 4.15E-04 | 2 |
| chr4:778661-780592 | CPLX1 | -0.12 | 4.30E-04 | 2 |
| chr1:6685071-6685691 | PHF13 | 0.11 | 4.93E-04 | 2 |
| chr7:96653467-96654199 | DLX5 | -0.12 | 4.96E-04 | 3 |
| chr16:88569267-88569491 | ZFPM1 | 0.12 | 5.17E-04 | 2 |
| chr16:88564052-88564394 | ZFPM1 | 0.14 | 5.31E-04 | 4 |
| chr5:10307520-10307913 | CMBL | 0.14 | 5.31E-04 | 2 |
| chr17:46629553-46629816 | HOXB3 | 0.11 | 5.63E-04 | 2 |
| chr7:87849003-87849594 | SRI | -0.15 | 6.10E-04 | 2 |
| chr6:29600192-29600661 | GABBR1 | 0.11 | 6.27E-04 | 3 |
| chr2:241827777-241828031 | C2orf54 | 0.11 | 6.28E-04 | 2 |
| chr10:105452338-105453230 | SH3PXD2A | -0.12 | 6.48E-04 | 2 |
| chr3:5024760-5024967 | BHLHE40 | 0.13 | 6.48E-04 | 2 |
| chr4:40858884-40859162 | APBB2 | -0.14 | 6.81E-04 | 3 |
| chr19:13319268-13319789 | CACNA1A | -0.12 | 7.20E-04 | 2 |
| chr5:6737704-6738004 | POLS | 0.13 | 7.90E-04 | 2 |
| chr22:37420225-37420900 | MPST | 0.12 | 8.41E-04 | 2 |
| chr2:127817994-127818231 | BIN1 | 0.15 | 9.43E-04 | 4 |
| chr11:279072-281700 | NLRP6 | 0.11 | 1.01E-03 | 2 |
| chr7:100608736-100609793 | MUC12 | -0.12 | 1.39E-03 | 2 |
| chr6:33244677-33245554 | B3GALT4 | -0.12 | 1.49E-03 | 6 |
| chr17:73583838-73586337 | MYO15B | -0.12 | 1.63E-03 | 2 |
| chr6:32847498-32847846 | PPP1R2P1 | 0.10 | 1.69E-03 | 2 |
| chr2:219263098-219265556 | CTDSP1;MIR26B | 0.11 | 1.75E-03 | 2 |
| chr16:2014164-2015451 | SNHG9;SNORA64;RPS2;SNORA78 | -0.11 | 2.15E-03 | 2 |
| chr17:36452840-36453141 | MRPL45 | 0.11 | 2.24E-03 | 2 |
| chr15:60690708-60690930 | ANXA2 | -0.14 | 2.26E-03 | 2 |
| chr6:31323946-31325211 | HLA-B | -0.13 | 2.35E-03 | 2 |
| chr19:4328658-4328865 | STAP2 | -0.13 | 3.49E-03 | 2 |
| chr2:227662082-227664912 | IRS1 | 0.11 | 3.50E-03 | 4 |
| chr17:75883803-75884328 | FLJ45079 | 0.10 | 8.93E-03 | 2 |
| chr6:30038881-30039477 | RNF39 | 0.13 | 1.13E-02 | 6 |
| chr13:30077245-30077490 | MTUS2 | -0.11 | 1.49E-02 | 2 |
| chr19:6752515-6753657 | SH2D3A | 0.13 | 1.50E-02 | 2 |
