# Supplementary material for: Differentially methylated loci in NAFLD cirrhosis are associated with key signaling pathways
Source: Clin Epigenetics. 2018 Jul 13;10:93. doi: 10.1186/s13148-018-0525-9 (PMC6044005; doi:10.1186/s13148-018-0525-9)
Supplement: Supplementary file 2 — Table S2. Top 100 differentially methylated CpG sites (DOCX 30 kb) [file 13148_2018_525_MOESM2_ESM.docx]

| **Table S2. Top 100 differentially methylated CpG sites** | | | | | | |
| --- | --- | --- | --- | --- | --- | --- |
|  |  | **Avg beta** | |  |  |  |
| **SiteID** | **Gene** | **Fibrosis** | **Normal** | **Δbeta** | **pvalue** | **FDR** |
| cg03206537 | CTSA | 0.490975236 | 0.594239567 | -0.10326433 | 1.0933E-09 | 8.46256E-05 |
| cg18708013 | ZC3H12D | 0.418927858 | 0.518964904 | -0.100037046 | 9.50286E-10 | 8.46256E-05 |
| cg19164131 | CAMTA1 | 0.552965891 | 0.44134798 | 0.111617911 | 4.77224E-09 | 0.000132972 |
| cg25343618 |  | 0.42130484 | 0.548744822 | -0.127439983 | 9.92807E-09 | 0.000132972 |
| cg00524374 |  | 0.332815702 | 0.5647602 | -0.231944499 | 8.19456E-09 | 0.000132972 |
| cg10465028 | OTUB1 | 0.496708942 | 0.628184446 | -0.131475505 | 6.62994E-09 | 0.000132972 |
| cg19544441 | CORO1B | 0.414226971 | 0.579804481 | -0.165577511 | 9.24672E-09 | 0.000132972 |
| cg14553705 |  | 0.450168454 | 0.627073719 | -0.176905265 | 4.28338E-09 | 0.000132972 |
| cg19607021 |  | 0.486593383 | 0.656336888 | -0.169743505 | 1.2598E-08 | 0.000132972 |
| cg00089453 | SYNPO | 0.468174694 | 0.583109452 | -0.114934758 | 2.39035E-09 | 0.000132972 |
| cg04822621 | LCP2 | 0.376445496 | 0.507555976 | -0.131110481 | 5.60152E-09 | 0.000132972 |
| cg07904452 |  | 0.427799125 | 0.636467127 | -0.208668001 | 5.74787E-09 | 0.000132972 |
| cg09824603 |  | 0.47043569 | 0.643131825 | -0.172696136 | 1.25639E-08 | 0.000132972 |
| cg08251901 | ARHGDIB | 0.438182882 | 0.605891335 | -0.167708453 | 1.67708E-08 | 0.000147668 |
| cg19057001 | ANXA6 | 0.469423519 | 0.617750229 | -0.14832671 | 1.81379E-08 | 0.000150422 |
| cg12123019 | IL12RB1 | 0.394342336 | 0.525768293 | -0.131425957 | 2.18098E-08 | 0.000166048 |
| cg05229759 | UBASH3B | 0.464932225 | 0.584021656 | -0.119089431 | 2.52424E-08 | 0.000166099 |
| cg18125510 | WARS;WDR25 | 0.385290655 | 0.544742087 | -0.159451432 | 2.3956E-08 | 0.000166099 |
| cg09356916 |  | 0.393477214 | 0.52689045 | -0.133413235 | 2.40664E-08 | 0.000166099 |
| cg13547889 | NOTCH1 | 0.451941476 | 0.555757905 | -0.10381643 | 2.24992E-08 | 0.000166099 |
| cg03813377 |  | 0.396394154 | 0.524616563 | -0.12822241 | 2.61398E-08 | 0.000168609 |
| cg03457485 | OTUB1 | 0.392039659 | 0.548850911 | -0.156811252 | 2.9984E-08 | 0.000169819 |
| cg23497569 |  | 0.596507411 | 0.475694621 | 0.12081279 | 3.29786E-08 | 0.000170708 |
| cg03909849 | ACTG1 | 0.358842327 | 0.53212006 | -0.173277733 | 3.69744E-08 | 0.000170708 |
| cg22699620 | ITGB2 | 0.496678109 | 0.638549467 | -0.141871358 | 4.20478E-08 | 0.000170708 |
| cg23047271 | PRICKLE2 | 0.414849185 | 0.567929677 | -0.153080492 | 3.23861E-08 | 0.000170708 |
| cg00741986 | TNIP2 | 0.486239497 | 0.626325073 | -0.140085575 | 3.30275E-08 | 0.000170708 |
| cg03030757 | FANCE | 0.400708572 | 0.540399982 | -0.13969141 | 3.7421E-08 | 0.000170708 |
| cg18739537 | NFKBIE | 0.337055076 | 0.543472518 | -0.206417442 | 3.8474E-08 | 0.000170708 |
| cg24921221 | LONRF1 | 0.32818615 | 0.50904773 | -0.18086158 | 3.47381E-08 | 0.000170708 |
| cg00109344 | RGS3 | 0.421950888 | 0.540475848 | -0.11852496 | 4.47937E-08 | 0.000173359 |
| cg08190450 | NXN | 0.435077418 | 0.584794053 | -0.149716635 | 4.754E-08 | 0.000178053 |
| cg07339236 | ATP9A | 0.495151027 | 0.655888453 | -0.160737426 | 4.82894E-08 | 0.000179413 |
| cg00860712 | BTG2 | 0.432574462 | 0.54683956 | -0.114265097 | 4.90807E-08 | 0.000179514 |
| cg05766107 |  | 0.464979954 | 0.590809728 | -0.125829774 | 5.45013E-08 | 0.000179514 |
| cg07499182 | STARD13 | 0.373400236 | 0.566923474 | -0.193523238 | 5.27044E-08 | 0.000179514 |
| cg05548488 | MAPKAPK3 | 0.456185598 | 0.561541508 | -0.105355911 | 5.29812E-08 | 0.000179514 |
| cg20465207 | ARHGEF2 | 0.324477079 | 0.524593017 | -0.200115938 | 5.61351E-08 | 0.000179557 |
| cg19736117 |  | 0.395809473 | 0.504656607 | -0.108847134 | 5.69566E-08 | 0.000179944 |
| cg19734536 | SLAMF1 | 0.336084508 | 0.520215137 | -0.184130629 | 6.07617E-08 | 0.000189012 |
| cg13052638 |  | 0.397806481 | 0.567630941 | -0.16982446 | 6.38664E-08 | 0.000192019 |
| cg17616646 | PITPNM1 | 0.413153632 | 0.550873833 | -0.137720201 | 6.57266E-08 | 0.000192019 |
| cg11171825 | PEX6 | 0.45359861 | 0.610749554 | -0.157150944 | 6.6233E-08 | 0.000192019 |
| cg13157980 | EIF2C2 | 0.361430474 | 0.515342646 | -0.153912172 | 6.34417E-08 | 0.000192019 |
| cg23683800 |  | 0.329702287 | 0.516766185 | -0.187063898 | 7.54096E-08 | 0.000202727 |
| cg14064762 | TRAF1 | 0.49988962 | 0.677411162 | -0.177521541 | 7.48221E-08 | 0.000202727 |
| cg10928294 | MEOX1 | 0.484406636 | 0.61515196 | -0.130745324 | 8.06597E-08 | 0.000209274 |
| cg08065733 | INHBB | 0.414590056 | 0.521180198 | -0.106590141 | 8.25684E-08 | 0.000211859 |
| cg01799671 | CMIP | 0.408506109 | 0.590003407 | -0.181497298 | 8.36468E-08 | 0.000212811 |
| cg10893986 | PRDM16 | 0.422270407 | 0.545186992 | -0.122916586 | 8.76527E-08 | 0.000214363 |
| cg27361227 | ARSB | 0.573989552 | 0.414599928 | 0.159389624 | 9.55252E-08 | 0.000219623 |
| cg21196487 | S100A2 | 0.448270584 | 0.557392947 | -0.109122363 | 1.21313E-07 | 0.000222811 |
| cg09331409 | MAP6 | 0.45127098 | 0.598817435 | -0.147546455 | 1.22155E-07 | 0.000222811 |
| cg10167235 | WARS | 0.423783409 | 0.563444947 | -0.139661538 | 9.98956E-08 | 0.000222811 |
| cg11621113 | MAN2B1;MORG1 | 0.473188436 | 0.613061127 | -0.13987269 | 1.14204E-07 | 0.000222811 |
| cg21335378 |  | 0.49472932 | 0.595592338 | -0.100863018 | 1.20916E-07 | 0.000222811 |
| cg22609522 | LOC730668 | 0.365783518 | 0.508363613 | -0.142580095 | 1.22339E-07 | 0.000222811 |
| cg13702846 | CCBP2 | 0.39608095 | 0.504670297 | -0.108589347 | 1.11114E-07 | 0.000222811 |
| cg03929796 | ALAS1 | 0.467531667 | 0.575445007 | -0.10791334 | 1.10423E-07 | 0.000222811 |
| cg06092869 |  | 0.464926604 | 0.578398958 | -0.113472354 | 1.2321E-07 | 0.000223094 |
| cg11784631 | NRM | 0.499642674 | 0.613209175 | -0.113566502 | 1.24711E-07 | 0.000223094 |
| cg02064267 |  | 0.402248778 | 0.518193846 | -0.115945069 | 1.28436E-07 | 0.000225702 |
| cg27292264 | MIR1280;EEFSEC | 0.401725791 | 0.517540354 | -0.115814563 | 1.28786E-07 | 0.000225702 |
| cg10023454 | QSOX1 | 0.625278999 | 0.486990449 | 0.138288551 | 1.34066E-07 | 0.00022961 |
| cg02732915 | PRDM8 | 0.593716317 | 0.47095516 | 0.122761157 | 1.34178E-07 | 0.00022961 |
| cg17130457 |  | 0.435653497 | 0.58202658 | -0.146373083 | 1.3764E-07 | 0.000230168 |
| cg00084094 | CORO1C | 0.389124342 | 0.504185989 | -0.115061647 | 1.3892E-07 | 0.000231245 |
| cg06160853 |  | 0.519358922 | 0.362663197 | 0.156695725 | 1.40879E-07 | 0.000231464 |
| cg07094298 | TNIP2 | 0.328091647 | 0.519188005 | -0.191096359 | 1.43508E-07 | 0.000232224 |
| cg22827210 | CHST11 | 0.451402821 | 0.560370491 | -0.108967671 | 1.4739E-07 | 0.00023539 |
| cg19323289 | NAV2 | 0.497759927 | 0.652129064 | -0.154369137 | 1.51067E-07 | 0.000237825 |
| cg02072495 | ANXA2 | 0.308968055 | 0.502752287 | -0.193784232 | 1.5811E-07 | 0.000242342 |
| cg07119067 |  | 0.456142068 | 0.57491027 | -0.118768202 | 1.63031E-07 | 0.00024456 |
| cg07340025 |  | 0.463289776 | 0.664059237 | -0.200769461 | 1.63392E-07 | 0.00024456 |
| cg11528914 | LCP2 | 0.489032252 | 0.607932388 | -0.118900136 | 1.67525E-07 | 0.00024456 |
| cg01798786 |  | 0.413968355 | 0.522224973 | -0.108256619 | 1.72858E-07 | 0.000244847 |
| cg03699074 | FAM38A | 0.392899355 | 0.58095856 | -0.188059205 | 1.79701E-07 | 0.000245708 |
| cg18263166 |  | 0.44315433 | 0.602594177 | -0.159439848 | 1.82501E-07 | 0.000247105 |
| cg10189135 | ATP6V0D1 | 0.438033818 | 0.553553413 | -0.115519595 | 1.83201E-07 | 0.000247332 |
| cg08504662 | ZNF497 | 0.470891873 | 0.576836547 | -0.105944674 | 1.93614E-07 | 0.000254006 |
| cg09541576 | C2orf34 | 0.622694346 | 0.484207719 | 0.138486627 | 1.97865E-07 | 0.000255429 |
| cg24876187 | TCF19;CCHCR1 | 0.419924036 | 0.531070761 | -0.111146724 | 1.98548E-07 | 0.000255429 |
| cg25063165 |  | 0.413645663 | 0.553655614 | -0.140009951 | 2.02345E-07 | 0.000258855 |
| cg20468939 | PKN1 | 0.481154418 | 0.602248967 | -0.121094548 | 2.0575E-07 | 0.000258955 |
| cg07739369 |  | 0.481293455 | 0.62128342 | -0.139989965 | 2.14355E-07 | 0.000260171 |
| cg19769080 | PPM1B | 0.459757785 | 0.61249333 | -0.152735545 | 2.14456E-07 | 0.000260171 |
| cg12093662 | ARHGAP25 | 0.423071162 | 0.523180852 | -0.10010969 | 2.10725E-07 | 0.000260171 |
| cg19617080 | ATP11B | 0.371712893 | 0.558173464 | -0.186460571 | 2.11137E-07 | 0.000260171 |
| cg15129183 | LOC257358 | 0.491380978 | 0.608189463 | -0.116808485 | 2.07947E-07 | 0.000260171 |
| cg01602153 | FAM110B | 0.488807073 | 0.606149643 | -0.11734257 | 2.12629E-07 | 0.000260171 |
| cg26546113 | DHX9 | 0.444226352 | 0.575335172 | -0.13110882 | 2.25644E-07 | 0.000263078 |
| cg02481934 | HIST1H3G | 0.412766094 | 0.544951753 | -0.132185659 | 2.3217E-07 | 0.000265578 |
| cg01668099 |  | 0.422055381 | 0.536438952 | -0.11438357 | 2.38E-07 | 0.00027025 |
| cg15646741 |  | 0.365792939 | 0.504226139 | -0.1384332 | 2.55418E-07 | 0.000274273 |
| cg10255535 |  | 0.412006387 | 0.523261621 | -0.111255234 | 2.56736E-07 | 0.000274732 |
| cg08040115 |  | 0.403269845 | 0.535840573 | -0.132570728 | 2.64331E-07 | 0.000279002 |
| cg04456219 |  | 0.487388293 | 0.68825402 | -0.200865728 | 2.65405E-07 | 0.0002795 |
| cg21761853 | FLOT1 | 0.466710532 | 0.573812388 | -0.107101856 | 2.67209E-07 | 0.000279684 |
| cg22245273 | TMIGD2 | 0.490317827 | 0.599017407 | -0.108699579 | 2.73846E-07 | 0.000281371 |
| cg07533239 | PLEKHG6 | 0.400363582 | 0.572518863 | -0.172155281 | 2.7822E-07 | 0.000282162 |
