# Supplementary figures and images for: Differentially methylated loci in NAFLD cirrhosis are associated with key signaling pathways
Source: Clin Epigenetics. 2018 Jul 13;10:93. doi: 10.1186/s13148-018-0525-9 (PMC6044005; doi:10.1186/s13148-018-0525-9)

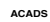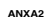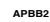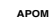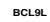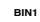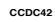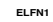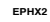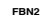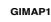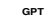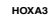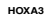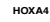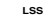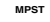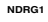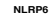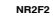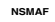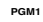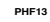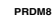

RALGAP2

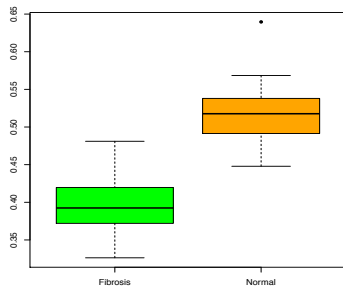

RASGEF1A

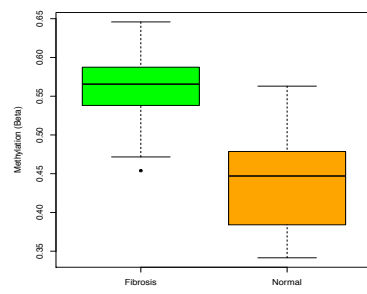

RNF39

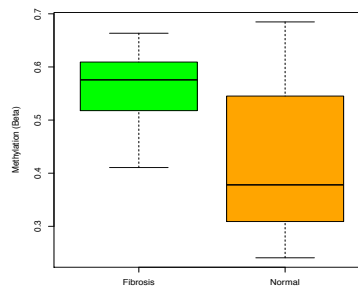

RNF182

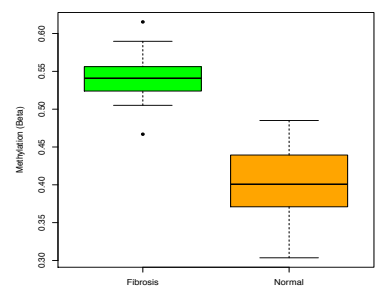

SH2D3A

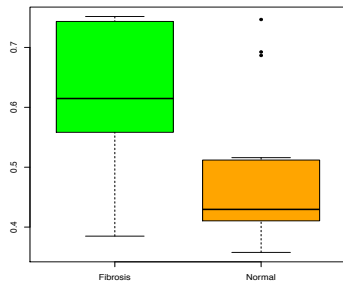

SHANK2

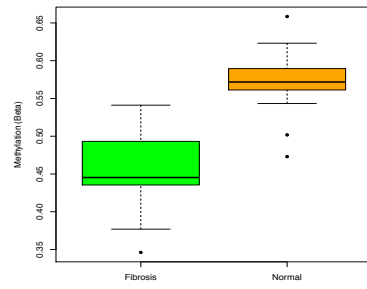

SHANK2

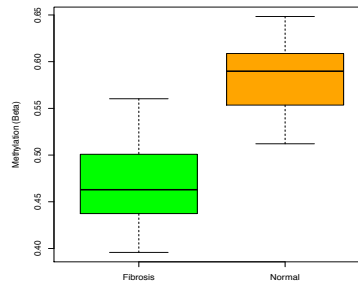

TOLLIP

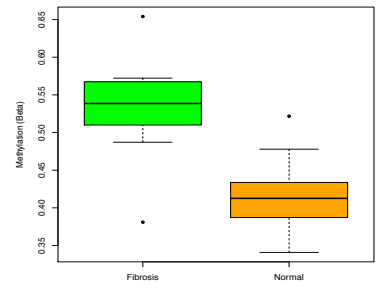

ZNF532

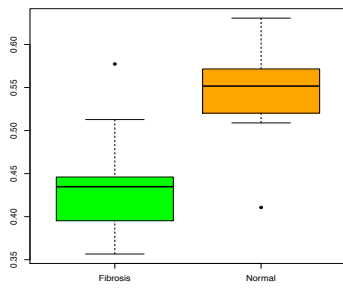

ZNF782

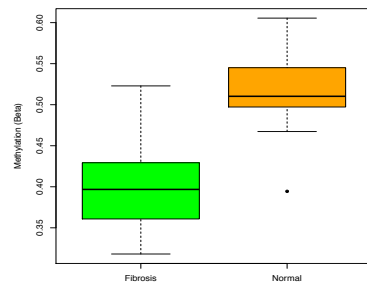

Supplement: Supplementary file 3 — Figure S1. Box plot of mean (± standard deviation) methylation β values for CGI associated with gene expression differences in patients with NAFLD fibrosis (n = 11) compared to individuals with normal liver histology (n = 15). (PDF 544 kb) [file 13148_2018_525_MOESM3_ESM.pdf]
