# Supplementary material for: Differentially methylated loci in NAFLD cirrhosis are associated with key signaling pathways
Source: Clin Epigenetics. 2018 Jul 13;10:93. doi: 10.1186/s13148-018-0525-9 (PMC6044005; doi:10.1186/s13148-018-0525-9)
Supplement: Supplementary file 4 — Table S3. Canonical pathways identified by pathway analysis using all significant CpG islands (N = 208). (DOCX 26 kb) [file 13148_2018_525_MOESM4_ESM.docx]

|  | | | |
| --- | --- | --- | --- |
| **Table S3. Canonical pathways identified by pathway analysis using all significant CpG islands (N=208)** | | | |
| **Ingenuity Canonical Pathways** | **FDR** | **Ratio** | **Molecules** |
| Production of Nitric Oxide and Reactive Oxygen Species in Macrophages | 1.41E-05 | 0.05 | RHOG,APOB,PPP1R3C,RHOB,MAP3K8,IRS1,RBP4,CYBA,APOM,PIK3CG |
| LXR/RXR Activation | 1.74E-05 | 0.07 | NCOR2,APOB,TF,VTN,RXRA,RBP4,PON3,APOM |
| FXR/RXR Activation | 2.34E-05 | 0.06 | APOB,TF,VTN,RXRA,RBP4,NR5A2,PON3,APOM |
| RAR Activation | 7.24E-05 | 0.05 | NCOR2,ADCY3,DUSP1,ZBTB16,RXRA,RBP4,ADCY9,NR2F2,PIK3CG |
| Alanine Biosynthesis II | 8.51E-05 | 1.00 | GPT2,GPT |
| Alanine Degradation III | 8.51E-05 | 1.00 | GPT2,GPT |
| Sphingosine-1-phosphate Signaling | 1.66E-04 | 0.06 | RHOG,ADCY3,PTK2B,RHOB,IRS1,ADCY9,PIK3CG |
| Phagosome Formation | 2.14E-04 | 0.05 | RHOG,INPP5D,VTN,RHOB,IRS1,ITGB2,PIK3CG |
| B Cell Receptor Signaling | 4.27E-04 | 0.04 | INPP5D,PTK2B,EBF1,MAP3K8,IRS1,ETS1,RASSF5,PIK3CG |
| IL-12 Signaling and Production in Macrophages | 4.37E-04 | 0.05 | APOB,MAP3K8,RXRA,IRS1,RBP4,APOM,PIK3CG |
| Glioma Invasiveness Signaling | 4.79E-04 | 0.07 | RHOG,VTN,RHOB,IRS1,PIK3CG |
| Acetate Conversion to Acetyl-CoA | 5.01E-04 | 0.50 | ACSS1,ACSL1 |
| Clathrin-mediated Endocytosis Signaling | 5.50E-04 | 0.04 | APOB,TF,ACTG1,IRS1,ITGB2,RBP4,APOM,PIK3CG |
| Signaling by Rho Family GTPases | 6.03E-04 | 0.04 | BAIAP2,RHOG,ACTG1,PKN1,PTK2B,RHOB,ARHGEF2,IRS1,PIK3CG |
| Thrombin Signaling | 6.61E-04 | 0.04 | RHOG,ADCY3,RHOB,ARHGEF2,IRS1,F2RL3,ADCY9,PIK3CG |
| Leukocyte Extravasation Signaling | 8.13E-04 | 0.04 | ACTG1,PTK2B,IRS1,ITGB2,CLDN1,CYBA,RASSF5,PIK3CG |
| G-Protein Coupled Receptor Signaling | 1.12E-03 | 0.03 | ADCY3,DUSP1,PTK2B,GABBR1,MAP3K8,IRS1,RGS12,ADCY9,PIK3CG |
| Type II Diabetes Mellitus Signaling | 1.26E-03 | 0.05 | ACSL1,SLC2A2,IRS1,PKM,NSMAF,PIK3CG |
| IL-4 Signaling | 1.45E-03 | 0.06 | INPP5D,HLA-DOA,IRS1,HLA-B,PIK3CG |
| Cardiac Hypertrophy Signaling | 1.66E-03 | 0.03 | RHOG,ADCY3,RHOB,MAP3K8,IRS1,CACNA1A,ADCY9,PIK3CG |
| ILK Signaling | 2.45E-03 | 0.04 | RHOG,ACTG1,RHOB,IRS1,LIMS2,ITGB2,PIK3CG |
| Virus Entry via Endocytic Pathways | 2.63E-03 | 0.05 | ACTG1,IRS1,HLA-B,ITGB2,PIK3CG |
| GABA Receptor Signaling | 3.89E-03 | 0.06 | ADCY3,GABBR1,SLC6A1,ADCY9 |
| Paxillin Signaling | 4.07E-03 | 0.04 | ACTG1,PTK2B,IRS1,ITGB2,PIK3CG |
| MSP-RON Signaling Pathway | 4.47E-03 | 0.06 | ACTG1,IRS1,ITGB2,PIK3CG |
| CXCR4 Signaling | 4.47E-03 | 0.04 | RHOG,ADCY3,RHOB,IRS1,ADCY9,PIK3CG |
| Rac Signaling | 4.68E-03 | 0.04 | BAIAP2,PTK2B,TIAM1,IRS1,PIK3CG |
| Tec Kinase Signaling | 5.13E-03 | 0.04 | RHOG,ACTG1,PTK2B,RHOB,IRS1,PIK3CG |
| Renin-Angiotensin Signaling | 5.62E-03 | 0.04 | ADCY3,PTK2B,IRS1,ADCY9,PIK3CG |
| Germ Cell-Sertoli Cell Junction Signaling | 5.62E-03 | 0.03 | RHOG,ACTG1,RHOB,MAP3K8,IRS1,PIK3CG |
| Non-Small Cell Lung Cancer Signaling | 5.75E-03 | 0.05 | RXRA,IRS1,RASSF5,PIK3CG |
| iCOS-iCOSL Signaling in T Helper Cells | 5.89E-03 | 0.04 | INPP5D,HLA-DOA,IRS1,HLA-B,PIK3CG |
| Cholesterol Biosynthesis I | 6.17E-03 | 0.15 | LSS,DHCR24 |
| Cholesterol Biosynthesis II (via 24,25-dihydrolanosterol) | 6.17E-03 | 0.15 | LSS,DHCR24 |
| Cholesterol Biosynthesis III (via Desmosterol) | 6.17E-03 | 0.15 | LSS,DHCR24 |
| Stearate Biosynthesis I (Animals) | 7.41E-03 | 0.07 | ACSL1,DHCR24,CYP2E1 |
| Small Cell Lung Cancer Signaling | 8.13E-03 | 0.05 | RXRA,IRS1,TRAF1,PIK3CG |
| PKCθ Signaling in T Lymphocytes | 8.13E-03 | 0.04 | HLA-DOA,MAP3K8,IRS1,HLA-B,PIK3CG |
| Th1 Pathway | 8.51E-03 | 0.04 | HLA-DOA,IRS1,HLA-B,ITGB2,PIK3CG |
| Leptin Signaling in Obesity | 8.51E-03 | 0.05 | ADCY3,IRS1,ADCY9,PIK3CG |
| Lanosterol Biosynthesis | 9.33E-03 | 1.00 | LSS, |
| Gap Junction Signaling | 9.77E-03 | 0.03 | GJB2,ADCY3,ACTG1,IRS1,ADCY9,PIK3CG |
| IL-8 Signaling | 1.02E-02 | 0.03 | RHOG,PTK2B,RHOB,IRS1,ITGB2,PIK3CG |
| Reelin Signaling in Neurons | 1.07E-02 | 0.04 | ARHGEF2,IRS1,ITGB2,PIK3CG |
| ERK/MAPK Signaling | 1.10E-02 | 0.03 | PPP1R3C,DUSP1,PTK2B,IRS1,ETS1,PIK3CG |
| Fcγ Receptor-mediated Phagocytosis in Macrophages and Monocytes | 1.10E-02 | 0.04 | INPP5D,ACTG1,PTK2B,PIK3CG |
| mTOR Signaling | 1.12E-02 | 0.03 | RHOG,RPS6,RHOB,IRS1,RPS2,PIK3CG |
| Breast Cancer Regulation by Stathmin1 | 1.23E-02 | 0.03 | ADCY3,PPP1R3C,ARHGEF2,IRS1,ADCY9,PIK3CG |
| Melanocyte Development and Pigmentation Signaling | 1.32E-02 | 0.04 | ADCY3,IRS1,ADCY9,PIK3CG |
| TR/RXR Activation | 1.32E-02 | 0.04 | NCOR2,RXRA,IRS1,PIK3CG |
| FcγRIIB Signaling in B Lymphocytes | 1.32E-02 | 0.06 | INPP5D,IRS1,PIK3CG |
| Th2 Pathway | 1.32E-02 | 0.03 | HLA-DOA,IRS1,HLA-B,ITGB2,PIK3CG |
| CDK5 Signaling | 1.35E-02 | 0.04 | ADCY3,PPP1R3C,CACNA1A,ADCY9 |
| Actin Nucleation by ARP-WASP Complex | 1.51E-02 | 0.05 | BAIAP2,RHOG,RHOB |
| RANK Signaling in Osteoclasts | 1.51E-02 | 0.04 | PTK2B,MAP3K8,IRS1,PIK3CG |
| VEGF Signaling | 1.55E-02 | 0.04 | ACTG1,PTK2B,IRS1,PIK3CG |
| Maturity Onset Diabetes of Young (MODY) Signaling | 1.58E-02 | 0.10 | SLC2A2,CACNA1A |
| Oxidative Ethanol Degradation III | 1.58E-02 | 0.10 | ACSS1,ACSL1 |
| Integrin Signaling | 1.66E-02 | 0.03 | RHOG,ACTG1,RHOB,IRS1,ITGB2,PIK3CG |
| Nur77 Signaling in T Lymphocytes | 1.74E-02 | 0.05 | HLA-DOA,RXRA,HLA-B |
| Gαq Signaling | 1.74E-02 | 0.03 | RHOG,PTK2B,RHOB,IRS1,PIK3CG |
| Glycine Biosynthesis I | 1.86E-02 | 0.50 | SHMT1, |
| L-cysteine Degradation III | 1.86E-02 | 0.50 | MPST, |
| Telomerase Signaling | 2.00E-02 | 0.04 | IRS1,ETS1,HDAC7,PIK3CG |
| IL-2 Signaling | 2.14E-02 | 0.05 | PTK2B,IRS1,PIK3CG |
| Pyridoxal 5'-phosphate Salvage Pathway | 2.24E-02 | 0.05 | GRK5,PKN1,MAP3K8 |
| HGF Signaling | 2.24E-02 | 0.03 | MAP3K8,IRS1,ETS1,PIK3CG |
| Ethanol Degradation IV | 2.24E-02 | 0.08 | ACSS1,ACSL1 |
| Lymphotoxin β Receptor Signaling | 2.45E-02 | 0.04 | IRS1,TRAF1,PIK3CG |
| Phospholipase C Signaling | 2.51E-02 | 0.02 | RHOG,ADCY3,RHOB,ARHGEF2,ADCY9,HDAC7 |
| Gαi Signaling | 2.57E-02 | 0.03 | ADCY3,GABBR1,RGS12,ADCY9 |
| PPARα/RXRα Activation | 2.63E-02 | 0.03 | NCOR2,ADCY3,RXRA,IRS1,ADCY9 |
| NGF Signaling | 2.63E-02 | 0.03 | RHOG,MAP3K8,IRS1,PIK3CG |
| Natural Killer Cell Signaling | 2.69E-02 | 0.03 | INPP5D,SH3BP2,IRS1,PIK3CG |
| Superpathway of Cholesterol Biosynthesis | 2.75E-02 | 0.07 | LSS,DHCR24 |
| Thiosulfate Disproportionation III (Rhodanese) | 2.75E-02 | 0.33 | MPST, |
| RhoA Signaling | 2.82E-02 | 0.03 | BAIAP2,ACTG1,PKN1,PTK2B |
| Colorectal Cancer Metastasis Signaling | 2.82E-02 | 0.02 | RHOG,ADCY3,RHOB,IRS1,ADCY9,PIK3CG |
| Caveolar-mediated Endocytosis Signaling | 2.82E-02 | 0.04 | ACTG1,HLA-B,ITGB2 |
| Role of JAK1 and JAK3 in γc Cytokine Signaling | 2.82E-02 | 0.04 | PTK2B,IRS1,PIK3CG |
| Th1 and Th2 Activation Pathway | 2.95E-02 | 0.03 | HLA-DOA,IRS1,HLA-B,ITGB2,PIK3CG |
| GM-CSF Signaling | 3.02E-02 | 0.04 | IRS1,ETS1,PIK3CG |
| Atherosclerosis Signaling | 3.09E-02 | 0.03 | APOB,ITGB2,RBP4,APOM |
| NRF2-mediated Oxidative Stress Response | 3.39E-02 | 0.03 | HACD3,ACTG1,IRS1,GSTP1,PIK3CG |
| Role of NFAT in Cardiac Hypertrophy | 3.39E-02 | 0.03 | ADCY3,IRS1,ADCY9,HDAC7,PIK3CG |
| Cellular Effects of Sildenafil (Viagra) | 3.39E-02 | 0.03 | ADCY3,ACTG1,CACNA1A,ADCY9 |
| GDNF Family Ligand-Receptor Interactions | 3.39E-02 | 0.04 | IRS1,GFRA3,PIK3CG |
| Dopamine Receptor Signaling | 3.47E-02 | 0.04 | ADCY3,PPP1R3C,ADCY9 |
| CD28 Signaling in T Helper Cells | 3.47E-02 | 0.03 | HLA-DOA,IRS1,HLA-B,PIK3CG |
| p70S6K Signaling | 3.47E-02 | 0.03 | RPS6,IRS1,F2RL3,PIK3CG |
| HMGB1 Signaling | 3.55E-02 | 0.03 | RHOG,RHOB,IRS1,PIK3CG |
| Arginine Degradation I (Arginase Pathway) | 3.63E-02 | 0.25 | OAT, |
| Thymine Degradation | 3.63E-02 | 0.25 | UPB1, |
| Uracil Degradation II (Reductive) | 3.63E-02 | 0.25 | UPB1, |
| Adipogenesis pathway | 3.63E-02 | 0.03 | PLIN1,EBF1,NR2F2,HDAC7 |
| P2Y Purigenic Receptor Signaling Pathway | 3.63E-02 | 0.03 | ADCY3,IRS1,ADCY9,PIK3CG |
| Gα12/13 Signaling | 3.72E-02 | 0.03 | PTK2B,IRS1,F2RL3,PIK3CG |
| CD40 Signaling | 3.72E-02 | 0.04 | IRS1,TRAF1,PIK3CG |
| Macropinocytosis Signaling | 3.98E-02 | 0.04 | IRS1,ITGB2,PIK3CG |
| IL-3 Signaling | 4.17E-02 | 0.04 | INPP5D,IRS1,PIK3CG |
| Renal Cell Carcinoma Signaling | 4.17E-02 | 0.04 | IRS1,ETS1,PIK3CG |
| B Cell Development | 4.17E-02 | 0.06 | HLA-DOA,HLA-B |
| Cardiac β-adrenergic Signaling | 4.17E-02 | 0.03 | ADCY3,PPP1R3C,CACNA1A,ADCY9 |
| Insulin Receptor Signaling | 4.27E-02 | 0.03 | INPP5D,PPP1R3C,IRS1,PIK3CG |
| Ethanol Degradation II | 4.57E-02 | 0.05 | ACSS1,ACSL1 |
| FLT3 Signaling in Hematopoietic Progenitor Cells | 4.57E-02 | 0.03 | INPP5D,IRS1,PIK3CG |
| Folate Polyglutamylation | 4.57E-02 | 0.20 | SHMT1, |
| dTMP De Novo Biosynthesis | 4.57E-02 | 0.20 | SHMT1, |
| GNRH Signaling | 4.57E-02 | 0.03 | ADCY3,PTK2B,MAP3K8,ADCY9 |
| NF-κB Activation by Viruses | 4.68E-02 | 0.03 | IRS1,ITGB2,PIK3CG |
| Antigen Presentation Pathway | 4.79E-02 | 0.05 | HLA-DOA,HLA-B |
| HER-2 Signaling in Breast Cancer | 4.90E-02 | 0.03 | IRS1,ITGB2,PIK3CG |
