# Supplementary material for: Differentially methylated loci in NAFLD cirrhosis are associated with key signaling pathways
Source: Clin Epigenetics. 2018 Jul 13;10:93. doi: 10.1186/s13148-018-0525-9 (PMC6044005; doi:10.1186/s13148-018-0525-9)
Supplement: Supplementary file 5 — Table S4. Validation of previously reported methylation findings. (DOCX 34 kb) [file 13148_2018_525_MOESM5_ESM.docx]

|  | | | | | | | | |
| --- | --- | --- | --- | --- | --- | --- | --- | --- |
| **Table S4. Validation of previously reported methylation findings** | | | | | | | | |
| **Current study** | | | | | | **Murphy et al (17)*** | **De Mello et al (16)** | |
| **CpG site** | **Chr** | **Gene** | **ΔB** | **p-value** | **FDR** | **Direction** | **Difference ΔB (%)** | **FDR** |
| cg00068038 | 2 | CRIM1 | -0.16 | 5.50E-05 | 1.92E-03 | Hypomethylated | -12.24 | 2.85E-04 |
| cg00431549 | 12 | MGP | -0.11 | 3.13E-03 | 2.29E-02 | Hypomethylated | -8.16 | 7.76E-03 |
| cg00582941 | 20 | AHCY | 0.10 | 1.94E-04 | 3.67E-03 | Hypermethylated | 4.35 | 2.30E-02 |
| cg01016119 | 3 | ARHGEF3 | -0.14 | 9.02E-06 | 8.92E-04 | Hypomethylated | -4.98 | 4.61E-02 |
| cg01030178 | 8 | TRIM55 | 0.11 | 8.69E-04 | 9.32E-03 | Hypermethylated | 4.35 | 2.47E-02 |
| cg01242400 | 20 | ZGPAT | 0.11 | 3.41E-04 | 5.12E-03 | Hypermethylated | 4.50 | 4.74E-02 |
| cg01310473 | 7 | FGL2;CCDC146 | -0.16 | 3.48E-04 | 5.18E-03 | Hypomethylated | -9.91 | 3.19E-03 |
| cg01879083 | 5 | CMBL | 0.15 | 4.09E-03 | 2.77E-02 | Hypermethylated | 6.80 | 1.24E-02 |
| cg01957582 | 22 | YWHAH;C22orf24 | -0.13 | 4.59E-07 | 3.34E-04 | Hypomethylated | -5.41 | 4.01E-02 |
| cg02366931 | 5 | CMYA5 | 0.13 | 2.42E-05 | 1.33E-03 | Hypermethylated | 9.81 | 2.48E-02 |
| cg02572796 | 4 | SORBS2 | 0.14 | 1.42E-06 | 4.68E-04 | Hypermethylated | 6.21 | 3.33E-02 |
| cg03034696 | 2 | CALM2 | -0.15 | 4.69E-06 | 6.92E-04 | Hypomethylated | -6.62 | 1.22E-02 |
| cg03317505 | 5 | TMEM173 | -0.12 | 6.70E-07 | 3.67E-04 | Hypomethylated | -4.93 | 4.01E-02 |
| cg03322353 | 4 | MAML3 | 0.16 | 9.92E-05 | 2.56E-03 | Hypermethylated | 5.81 | 1.83E-02 |
| cg03761750 | 12 | VWF | -0.13 | 3.83E-04 | 5.50E-03 | Hypomethylated | -5.71 | 1.17E-02 |
| cg03851835 | 11 | SLC35F2 | -0.15 | 6.99E-05 | 2.15E-03 | Hypomethylated | -7.97 | 7.15E-04 |
| cg03983223 | 2 | WIPF1 | -0.13 | 4.50E-05 | 1.75E-03 | Hypomethylated | -4.76 | 1.69E-02 |
| cg04104489 | 1 | PGM1 | 0.15 | 1.00E-06 | 4.14E-04 | Hypermethylated | 7.18 | 1.52E-02 |
| cg04275847 | 10 | OAT | 0.15 | 2.35E-04 | 4.11E-03 | Hypermethylated | 6.25 | 2.30E-02 |
| cg04372674 | 7 | AQP1 | -0.15 | 3.48E-05 | 1.56E-03 | Hypomethylated | -6.46 | 2.80E-02 |
| cg04693046 | 7 | GPER;C7orf50 | 0.10 | 6.14E-04 | 7.42E-03 | Hypermethylated | 5.93 | 8.63E-04 |
| cg04806177 | 16 | SEPX1 | 0.16 | 4.72E-05 | 1.79E-03 | Hypermethylated | 5.69 | 1.87E-02 |
| cg05433222 | 7 | PON1 | 0.12 | 3.68E-05 | 1.60E-03 | Hypermethylated | 5.41 | 3.97E-02 |
| cg05626376 | 6 | DOM3Z;STK19 | 0.12 | 4.95E-05 | 1.82E-03 | Hypermethylated | 4.78 | 2.90E-02 |
| cg05911082 | 16 | SLC7A5 | 0.14 | 7.23E-04 | 8.25E-03 | Hypermethylated | 8.57 | 1.04E-04 |
| cg06186808 | 14 | FITM1 | 0.12 | 1.61E-05 | 1.12E-03 | Hypermethylated | 6.17 | 2.92E-04 |
| cg06248767 | 8 | LYN | -0.14 | 2.69E-03 | 2.05E-02 | Hypomethylated | -6.99 | 4.76E-04 |
| cg06635797 | 19 | UHRF1 | -0.16 | 5.93E-06 | 7.53E-04 | Hypomethylated | -7.01 | 1.70E-02 |
| cg07891271 | 15 | CHSY1 | -0.13 | 8.36E-07 | 3.98E-04 | Hypomethylated | -4.63 | 4.28E-02 |
| cg08110861 | 6 | APOM | 0.13 | 5.86E-04 | 7.19E-03 | Hypermethylated | 7.44 | 3.70E-03 |
| cg08130572 | 6 | FYN | -0.11 | 9.16E-06 | 8.97E-04 | Hypomethylated | -5.46 | 2.27E-02 |
| cg08656816 | 17 | CCL5 | -0.13 | 7.02E-06 | 8.03E-04 | Hypomethylated | -5.99 | 4.14E-02 |
| cg09038676 | 11 | GSTP1 | -0.14 | 5.52E-05 | 1.92E-03 | Hypomethylated | -7.43 | 2.82E-03 |
| cg09246479 | 22 | C22orf45;UPB1 | 0.13 | 6.41E-04 | 7.63E-03 | Hypermethylated | 5.58 | 1.08E-02 |
| cg09676669 | 7 | AQP1 | -0.13 | 4.95E-05 | 1.83E-03 | Hypomethylated | -7.43 | 7.65E-03 |
| cg09813248 | 12 | ACADS | 0.12 | 2.14E-04 | 3.88E-03 | Hypermethylated | 5.30 | 1.28E-02 |
| cg09962952 | 19 | SH2D3A | 0.15 | 5.82E-03 | 3.58E-02 | Hypermethylated | 15.65 | 4.24E-04 |
| cg10273135 | 5 | CMYA5 | 0.10 | 1.01E-04 | 2.58E-03 | Hypermethylated | 8.26 | 1.13E-02 |
| cg10425361 | 2 | CYS1 | -0.14 | 8.53E-05 | 2.37E-03 | Hypomethylated | -12.90 | 7.14E-05 |
| cg10572943 | 21 | IFNGR2 | -0.13 | 5.59E-06 | 7.37E-04 | Hypomethylated | -6.30 | 4.67E-03 |
| cg11153969 | 1 | C4BPA | 0.15 | 5.61E-05 | 1.94E-03 | Hypermethylated | 6.51 | 2.06E-02 |
| cg11706729 | 6 | PSMB8;TAP1 | -0.14 | 2.31E-06 | 5.40E-04 | Hypomethylated | -5.62 | 3.01E-02 |
| cg11827925 | 7 | AQP1 | -0.14 | 8.73E-04 | 9.35E-03 | Hypomethylated | -10.89 | 7.99E-04 |
| cg11982546 | 8 | LYN | -0.13 | 1.05E-04 | 2.64E-03 | Hypomethylated | -4.57 | 1.87E-02 |
| cg12100791 | 16 | PYCARD | -0.14 | 1.61E-06 | 4.87E-04 | Hypomethylated | -6.51 | 3.36E-02 |
| cg12275410 | 10 | ZNF511;TUBGCP2 | 0.11 | 1.95E-04 | 3.68E-03 | Hypermethylated | 4.30 | 3.93E-02 |
| cg12278705 | 1 | DDAH1;CYR61 | -0.12 | 3.28E-04 | 5.00E-03 | Hypomethylated | -5.61 | 3.40E-02 |
| cg12649208 | 17 | ACSF2 | 0.11 | 9.96E-05 | 2.56E-03 | Hypermethylated | 6.32 | 9.19E-03 |
| cg13781956 | 9 | TNC | -0.12 | 1.81E-06 | 5.00E-04 | Hypomethylated | -4.45 | 4.51E-02 |
| cg14035238 | 2 | WIPF1 | -0.12 | 3.26E-05 | 1.52E-03 | Hypomethylated | -4.56 | 1.69E-02 |
| cg14083603 | 20 | ZGPAT | 0.11 | 1.08E-03 | 1.08E-02 | Hypermethylated | 4.77 | 1.17E-02 |
| cg14666369 | 12 | ACADS | 0.18 | 8.82E-05 | 2.41E-03 | Hypermethylated | 9.03 | 3.19E-03 |
| cg14682080 | 5 | POLS | 0.11 | 5.85E-04 | 7.19E-03 | Hypermethylated | 8.10 | 9.95E-04 |
| cg14723423 | 19 | APOC2;APOC4 | 0.12 | 6.79E-04 | 7.91E-03 | Hypermethylated | 5.00 | 2.98E-02 |
| cg15223899 | 22 | C22orf45;UPB1 | 0.15 | 4.96E-05 | 1.83E-03 | Hypermethylated | 4.62 | 4.15E-02 |
| cg15373767 | 7 | AQP1 | -0.14 | 4.69E-05 | 1.78E-03 | Hypomethylated | -6.94 | 8.49E-03 |
| cg15565057 | 1 | FCAMR | -0.12 | 5.59E-07 | 3.52E-04 | Hypomethylated | -6.77 | 1.14E-02 |
| cg15882878 | 12 | RBP5;CLSTN3 | 0.13 | 6.65E-04 | 7.80E-03 | Hypermethylated | 5.80 | 3.10E-02 |
| cg16062483 | 14 | C14orf64 | -0.12 | 6.42E-03 | 3.85E-02 | Hypomethylated | -13.57 | 1.87E-04 |
| cg16107628 | 1 | TAGLN2 | -0.20 | 2.32E-06 | 5.42E-04 | Hypomethylated | -10.80 | 1.65E-03 |
| cg16861209 | 19 | SLC7A9 | 0.11 | 8.21E-04 | 8.98E-03 | Hypermethylated | 5.31 | 1.27E-02 |
| cg17977250 | 3 | GFM1;LXN | -0.10 | 5.84E-04 | 7.18E-03 | Hypomethylated | -7.07 | 7.13E-03 |
| cg18027683 | 1 | CYR61 | -0.11 | 2.57E-05 | 1.37E-03 | Hypomethylated | -6.47 | 1.62E-02 |
| cg18080604 | 7 | AQP1 | -0.13 | 1.43E-03 | 1.31E-02 | Hypomethylated | -11.37 | 5.36E-04 |
| cg18495047 | 22 | C22orf45;UPB1 | 0.12 | 1.28E-04 | 2.92E-03 | Hypermethylated | 4.72 | 7.03E-03 |
| cg18566515 | 10 | FGFR2 | -0.15 | 1.70E-04 | 3.41E-03 | Hypomethylated | -9.58 | 2.28E-03 |
| cg21436413 | 4 | SEL1L3 | -0.13 | 4.63E-04 | 6.19E-03 | Hypomethylated | -13.18 | 1.14E-03 |
| cg22164298 | 4 | ANK2 | -0.12 | 1.10E-05 | 9.59E-04 | Hypomethylated | -6.94 | 9.93E-04 |
| cg22689909 | 5 | GLRX | -0.13 | 1.80E-06 | 5.00E-04 | Hypomethylated | -5.29 | 2.50E-02 |
| cg22984132 | 7 | TMUB1;FASTK | 0.13 | 9.03E-04 | 9.57E-03 | Hypermethylated | 7.25 | 3.14E-03 |
| cg23013850 | 2 | FAM124B | 0.14 | 4.96E-04 | 6.47E-03 | Hypermethylated | 5.98 | 3.65E-02 |
| cg23239690 | 10 | MGMT | 0.14 | 6.02E-04 | 7.33E-03 | Hypermethylated | 8.47 | 4.71E-04 |
| cg23284609 | 5 | CMBL | 0.13 | 4.09E-05 | 1.68E-03 | Hypermethylated | 6.08 | 7.61E-03 |
| cg23654821 | 3 | CSRNP1 | -0.16 | 1.48E-05 | 1.08E-03 | Hypomethylated | -6.93 | 1.91E-02 |
| cg24154340 | 1 | CD48 | -0.10 | 8.44E-06 | 8.68E-04 | Hypomethylated | -5.02 | 9.31E-03 |
| cg24319651 | 12 | RBP5 | 0.13 | 2.49E-04 | 4.25E-03 | Hypermethylated | 6.26 | 2.32E-02 |
| cg24921614 | 7 | C7orf20 | 0.12 | 1.57E-03 | 1.40E-02 | Hypermethylated | 8.05 | 2.16E-03 |
| cg25075794 | 7 | AQP1 | -0.15 | 1.91E-05 | 1.20E-03 | Hypomethylated | -6.89 | 1.48E-02 |
| cg25455724 | 6 | APOM | 0.11 | 1.52E-04 | 3.21E-03 | Hypermethylated | 5.57 | 1.05E-02 |
| cg25727025 | 3 | GFM1;LXN | -0.11 | 2.00E-04 | 3.73E-03 | Hypomethylated | -4.94 | 7.71E-03 |
| cg25946605 | 2 | CYS1 | -0.13 | 3.95E-04 | 5.61E-03 | Hypomethylated | -9.75 | 5.07E-04 |
| cg26550235 | 1 | IFFO2 | -0.16 | 2.49E-05 | 1.35E-03 | Hypomethylated | -12.49 | 6.56E-03 |
| cg26662324 | 10 | CTBP2 | -0.16 | 4.84E-06 | 6.98E-04 | Hypomethylated | -5.13 | 9.43E-03 |
| cg26683425 | 14 | C14orf43 | -0.12 | 4.54E-06 | 6.83E-04 | Hypomethylated | -4.09 | 4.30E-02 |
| cg27040468 | 10 | BICC1 | -0.16 | 3.82E-04 | 5.49E-03 | Hypomethylated | -10.76 | 5.78E-03 |
| cg27126059 | 22 | C22orf45;UPB1 | 0.11 | 1.64E-04 | 3.35E-03 | Hypermethylated | 5.59 | 2.87E-03 |
| * only directional data were available for this study  The results from Ahrens et al (16) and Hotta et al (19) were not included because individual differentially methylated CpG sites or locations were not available. | | | | | | | | |
